# Thymidine Phosphorylase Promotes Abdominal Aortic Aneurysm via VSMC Modulation and Matrix Remodeling in Mice and Humans

**DOI:** 10.1155/cdr/1129181

**Published:** 2024-12-18

**Authors:** Liang Hong, Hong Yue, Dunpeng Cai, Autumn DeHart, Gretel Toloza-Alvarez, Lili Du, Xianwu Zhou, Xiaoping Fan, Huanlei Huang, Shiyou Chen, Shaik O. Rahaman, Jian Zhuang, Wei Li

**Affiliations:** ^1^Department of Cardiac Surgery, Guangdong Cardiovascular Institute, Guangdong Provincial People's Hospital, Guangdong Academy of Medical Sciences, Guangzhou, China; ^2^Department of Biomedical Sciences, Joan C. Edwards School of Medicine at Marshall University, Huntington, West Virginia, USA; ^3^Department of Surgery, University of Missouri School of Medicine, Columbia, Missouri, USA; ^4^Department of Cardiovascular Surgery, Guangdong Provincial People's Hospital (Guangdong Academy of Medical Sciences), Southern Medical University, Guangzhou, China; ^5^The Research Service, Harry S. Truman Memorial Veterans Hospital, Columbia, Missouri, USA; ^6^Department of Nutrition and Food Science, University of Maryland, College Park, Maryland, USA

**Keywords:** abdominal aortic aneurysm, inflammation, matrix metalloproteinase, thymidine phosphorylase, vascular smooth muscle cells

## Abstract

**Background:** Thymidine phosphorylase (TYMP) promotes platelet activation and thrombosis while suppressing vascular smooth muscle cell (VSMC) proliferation. Both processes are central to the development and progression of abdominal aortic aneurysms (AAAs). We hypothesize that TYMP plays a role in AAA development.

**Methods:** Male wild-type (WT) C57BL/6J and *Tymp^−/−^* mice, fed a Western diet (WD) (TD.88137), were subjected to the 4-week Ang II infusion–induced AAA model. AAA progression was monitored by echography and confirmed through necropsy. Whole-body inflammation was assessed using a plasma cytokine array. Mechanistic studies were conducted using TYMP-overexpressing rat VSMC cell lines and primary VSMCs cultured from WT and *Tymp^−/−^* mouse thoracic aortas. Histological studies were performed on human AAA and normal aorta samples.

**Results:** Elevated TYMP levels were observed in human AAA vessel walls. While WT mice exhibited a 28.6% prevalence of Ang II infusion–induced AAA formation, *Tymp^−/−^* mice were protected. TYMP enhanced MMP2 expression, secretion, and activation in VSMCs, which was inhibited by tipiracil, a selective TYMP inhibitor. Systemically, TYMP promoted proinflammatory cytokine expression, and its absence attenuated TNF-*α*-induced MMP2 and AKT activation. WT VSMCs treated with platelets lacking TYMP showed a higher proliferation rate than cells treated with WT platelets. Additionally, TYMP increased activated TGF*β*1 expression in cultured VSMCs and human AAA vessel walls. In WT VSMCs, TYMP augmented thrombospondin-1 type 1 repeat domain (TSR)–stimulated TGF*β*1 signaling, increasing connective tissue growth factor and MMP2 production. TSR also enhanced AKT activation in WT VSMCs but had the opposite effect in *Tymp^−/−^* cells. TSR-enhanced MMP2 activation in WT VSMCs was attenuated by LY294002 (a PI3K inhibitor) but not by SB431542 (a TGF*β*1 inhibitor); both inhibitors had indiscernible effects on *Tymp^−/−^* VSMC.

**Conclusion:** TYMP emerges as a novel regulatory force in vascular biology, influencing VSMC function and inflammatory responses to promote AAA development.

## 1. Introduction

Abdominal aortic aneurysm (AAA) is a serious vascular condition characterized by a localized dilation of the abdominal aorta (AA), particularly among older men. It is more prevalent in Western nations, presenting a substantial challenge to healthcare systems [1, 2]. Rupture of an AAA is often fatal in asymptomatic patients, contributing to at least 4.5 deaths per 1000 individuals [3]. The only effective treatment for symptomatic AAA patients is surgery, either through traditional open repair or endovascular grafting and stenting. However, these procedures are invasive, associated with significant complications, and require postoperative supportive care. Additionally, clinical studies have shown that these surgical interventions offer limited long-term benefits [4].

Several factors influence the risk of AAA, including age, male gender, hypertension, smoking, obesity, hyperlipidemia, and genetic disorders [4–6]. Additionally, a Western diet (WD), which enhances systemic low-grade inflammation, has been identified as a contributor to AAA development [7]. This observation suggests that children who frequently consume junk food or are obese could be at high risk for developing AAA in the future. However, there is currently no ideal animal model to effectively mimic these chronic conditions. The manifestation of AAA involves structural and functional changes in vascular smooth muscle cells (VSMCs), the extracellular matrix, elastin, and the accumulation of proinflammatory agents and cytokines. AAA progression can span decades and often remains asymptomatic. Currently, nonsurgical treatments for AAA are still in the exploratory phase and have not been firmly established. Moreover, no proven medical therapies are available to halt AAA progression once it has developed. Therefore, urgent research is needed to explore new cellular and molecular mechanisms that could lead to effective treatments.

The onset of AAA is driven by multiple genetic factors, including the low-density lipoprotein receptor, matrix metalloproteinases (MMPs), particularly MMP2 and MMP9, transforming growth factor-beta1 (TGF*β*1), and angiotensin II (Ang II) [8]. These factors contribute to the weakening of the aortic wall by promoting the loss of VSMCs and increasing extracellular matrix degradation in the tunica media, both of which are central to AAA pathogenesis. Thymidine phosphorylase (TYMP), also known as platelet-derived endothelial cell growth factor, is expressed by endothelial cells, platelets, and certain inflammatory cells. TYMP plays a significant role in platelet activation and thrombosis [9–11], enhances chemotaxis in endothelial cells, and inhibits VSMC proliferation [12–15]. Our previous research suggested a potential association between TYMP and MMP2/9 in the angiogenic processes [16]. However, the specific role of TYMP in the AAA environment remains unexplored. This study is aimed at investigating the hypothesis that TYMP, by enhancing MMP expression and activity in VSMCs and intensifying systemic inflammation, contributes to the weakening of the aortic wall, thereby promoting AAA development and progression.

## 2. Materials and Methods

### 2.1. Animals

This study utilized WT and *Tymp^−/−^* mice on a C57BL/6J background [11]. The power analysis was based on a previous study, in which 13 WT mice fed a regular laboratory diet and receiving Ang II infusion showed a 20% incidence of AAA formation [17]. We hypothesized that WD feeding would increase the incidence of AAA to an estimated 30% in WT mice. Assuming a 0% incidence in *Tymp^−/−^* mice, we calculated a minimal sample size of 21 per group, with an alpha of 0.5 and 80% power. Two sets of mice were subjected to the AAA model, resulting in a total of 26 mice per genotype.

The mice had ad libitum access to a standard laboratory rodent diet or WD after binding involved in the experiment and water. Given the significant influence of female sex hormones on vascular disease development [18], this study focused on male mice aged 4–16 weeks. Ethical approval was obtained from the Institutional Animal Care and Use Committee (IACUC) of Marshall University (IACUC#: 1033528, PI: Wei Li). All procedures adhered to the NIH Guide for the Care and Use of Laboratory Animals.

### 2.2. Establishment of AAA Model and Vascular Ultrasonography

We adopted the AAA model as mentioned by Daugherty and Cassis [19], with some modifications. Supporting Information 3: Figure S1 outlines the flow of the study. Briefly, mice were fed a WD (TD88137, Envigo) starting at 4 weeks of age for 8 weeks. Subsequently, mice received implantation of an Ang II-containing osmotic mini pump (Alzet, Model 2004), which delivered Ang II (Alomone labs, Cat# GPA-100) to mice at a dose of 1 *μ*g/kg/min for 4 weeks. Initial anesthesia was introduced in a small chamber with inhalation of 5% isoflurane (Attane, Inhalant Anesthetic, RXISO-250) mixed with 100% O_2_ at a flow rate of 1 L/min, delivered with a Harvard Apparatus Single Animal Tabletop Isoflurane Anesthesia System. The depth of anesthesia was confirmed by toe pinching. The mouse was then transferred onto a heating pad, and rectal temperature was maintained at 37°C, monitored with a rectal probe. Mouse anesthesia was maintained with 1.5%–2% isoflurane with 100% O_2_ via a facemask. The hair on the dorsal region of the neck was removed, and the skin was sanitized with Povidone Iodine Wipes followed by Alcohol Wipes. The mouse was covered with a sterilized surgical sheet with a 2 × 2 cm hole in the center to expose the surgical site. A small 1-cm incision was conducted on the dorsal neck, and a subcutaneous tunnel to the flank was created. The Ang II-containing osmotic pump was pushed into the flank through the subcutaneous tunnel. The incision was closed using interrupted sutures. Mice were administered subcutaneous buprenorphine (Buprenex, 0.05 mg/kg), twice per day, for pain control during the first 3 days and then as needed. Mice continued on the WD for an additional 4 weeks.

Vascular ultrasonography was performed on the first set of mice (14 WT and 13 *Tymp^−/−^*) to monitor the dynamic changes of the AA. We monitored the changes in the aortic inner diameter, blood flow, and other special findings such as thrombus formation, hematoma formation, obvious wall thickness, and calcification, before, 2 weeks, and 4 weeks after the minipump implantation. The inner diameter of the AA was measured at the suprarenal level. Flow parameters, including peek and mean velocity, time range of pulse, and acceleration and deceleration of pulse, which are affected by the aorta size or the presence of hematoma, were collected.

### 2.3. Gross Examination of AAA

Four weeks after chronic Ang II infusion, mice were anesthetized with isoflurane again, and laparotomy was carried out through the middle abdominal incision. Euthanasia was carried out by exsanguination, drawing whole blood through the inferior vena cava (IVC) puncture. Subsequently, mice were perfused with 10 mL of 10% formalin through the left ventricle to remove residual blood from the vessels. Abdominal organs and adipose tissues were then removed. If an aneurysm was visually confirmed, the soft tissues surrounding the aorta were cleaned, and a photograph of the AAA was taken. The entire aorta, including major branches, was isolated by careful dissection from surrounding tissues and fixed in 10% formalin for 48 h. The aorta with an aneurysm was sectioned into three segments and embedded in paraffin. In a separate group of mice (12 WT and 13 *Tymp^−/−^*), perfusion with cold phosphate-buffered saline (PBS) was performed, and aorta segments were directly embedded in optimal cutting temperature compound (OCT) for frozen sectioning. Sections of 6 *μ*m were cut and utilized for histological examination or in situ zymography.

### 2.4. Histology Examination

Hematoxylin and eosin (H&E), Elastica van Gieson (EVG) staining, and Masson's trichrome staining were performed to assess vascular structure, elastin integrity, and fibrosis [17, 20]. Standard immunohistochemical (IHC) or immunofluorescent staining was conducted using antibodies as detailed in the Results section.

As listed in Supporting Information 1: Table S1, a total of 16 AAA samples were utilized for histological examination: Eight were obtained from Chinese patients (#1–#8) at the Guangdong Cardiovascular Institute, and eight were obtained from Caucasian patients (#9–#16) at the University of Missouri School of Medicine. Patients with genetic disorders, such as Loyet–Dietz syndrome and Marfan syndrome, were excluded from this study. Normal human aortic samples harvested from organ donors were used as controls. The human AAA samples were carefully processed by removing peripheral tissues, and parts of the aneurysm tissues were fixed in formalin and embedded in paraffin. These samples were used to histologically analyze the expression of TYMP (12383-1-AP, Proteintech; sc-47702, Santa Cruz Biotechnology, Inc.), *α*-smooth muscle actin (*α*-SMA) (A2547, Millipore Sigma), phosphorylated AKT (p-AKT) (#4060, Cell Signaling), and TGF*β*1 (sc-130348, Santa Cruz; 21898-1-AP, Proteintech). Image scoring was performed based on representative images presented in Supporting Information 3: Figure S2.

The human studies were approved by the Institute Research Committee of Guangdong General Hospital (IRB#: GDREC2016255H, PI: Qiuxiong Lin) and by the Institutional Review Board of the University of Missouri School of Medicine (IRB#2026026, PI: Shiyou Chen). Written informed consent was obtained from all subjects. The investigation conformed to the principles outlined in the Declaration of Helsinki.

### 2.5. Quantitative Polymerase Chain Reaction (qPCR)

Total RNA was isolated from the human aortic vessel wall, frozen AAA samples (AAA #1–#8), healthy donors, or VSMCs using the Qiagen Universal RNA extraction kit. One microgram of total RNA was used for cDNA construction using the Super Script VILO cDNA Synthesis Kit (Thermofisher Scientific, Waltham, MA). Supporting Information 2: Table S2 lists the primers used for analyzing the genes targeted. The PowerUp SYBR Green Master Mix (ThermoFisher) kit was used for qPCR analysis using the ABI SimpliAmp Thermal Cycler. The qPCR products were validated using melting curves.

### 2.6. Cell Culture

A rat VSMC cell line, C2, stably expressing human TYMP, and the control cell line, PC, were established in previous studies [12, 14]. Additionally, VSMCs were cultured from the thoracic aorta of WT and *Tymp^−/−^* mice using an explant method [12], and cells from Passages 4 to 8 were used in this study. All cells were cultured in full culture media (FCM) composed of Dulbecco's modified Eagle's medium (DMEM), 10% fetal bovine serum, and antibiotics.

### 2.7. To Study the Impact of Platelets on VSMC Proliferation

Platelets were isolated from WT and *Tymp^−/−^* mice and washed to remove plasma components [10, 11, 21]. In brief, mice were anesthetized with ketamine/xylazine (100/10 mg/kg), and whole blood was drawn from the IVC using sterile 0.109 M sodium citrate as an anticoagulant. Tyrode's buffer (0.7 volume of the whole blood drawn) was added into the blood, which was then centrifuged at 100 × g for 10 min. The supernatant (platelet-rich plasma) was transferred into a new clean tube, and platelets were pelleted by centrifugation at 735 × g for 5 min in the presence of 100 nM PGE1. Platelets were resuspended in the PBS, and cell concentration was counted with a hematocytometer.

Murine WT VSMCs (1.00E + 03/well) were seeded into 96-well plates, serum-starved overnight, and then stimulated with FCM containing WT or *Tymp^−/−^* platelets (1.00E + 07 platelets/well). Cell proliferation was measured after 12 and 24 h using an MTT assay.

### 2.8. Impact of TYMP on MMP Expression and Activation in VSMCs

C2 and PC cells were cultured in FCM for 8 h, washed with PBS, and then incubated in serum-free DMEM for 18 h. The media were collected for gelatin zymography [16, 22]. Subsequently, the serum-starved cells were treated with serum-free DMEM containing tumor necrosis factor-*α* (TNF-*α*) (10 ng/mL) for various durations. Cells were lysed in RIPA buffer containing protease and phosphatase inhibitors for Western blot assay. In another set of experiments, serum-starved C2 and PC cells were treated with serum-free DMEM in the presence or absence of 1 *μ*M Ang II for 24 h. The culture media were collected for zymography, and the cells were lysed in RIPA buffer for Western blot assays. Additionally, serum-starved C2 and PC cells, or WT and *Tymp^−/−^* VSMCs, were treated with the thrombospondin-1 (TSP1) type 1 repeat domain (TSR) [23] in the presence or absence of LY294002 (a potent phosphoinositide 3-kinase, PI3K, inhibitor), SB431542 (a selective TGF*β*1 inhibitor), or tipiracil (a selective TYMP inhibitor) for various durations. Conditioned media were collected for zymography, while the cells were collected for Western blot analyses or qPCR.

### 2.9. In Situ Zymography

The AAs embedded in OCT were sectioned and mounted on a slide glass. The sections were washed with PBS and then incubated with a reaction buffer containing 25 *μ*g/mL fluorescein-conjugated DQ gelatin (D12054, ThermoFisher Scientific) for 18 h at room temperature in a dark, humid slide incubation box [24]. Images were captured under conditions that eliminated background autofluorescence, utilizing *Tymp^−/−^* sections incubated with reaction buffer only. The mean fluorescent intensity of each 20x image (green channel only), as well as the mean background intensity (without any tissues), was analyzed with ImageJ. The data were presented as the whole image mean minus the background mean.

### 2.10. Mouse Plasma Cytokine Array and Triglyceride (TG) Assay

Mouse plasma was pooled from randomly selected 6 WT mice (3 with AAA and 3 randomly selected) or 6 *Tymp^−/−^* mice (1 with AAA and 5 randomly selected). This pooled plasma was then used for determining plasma cytokine levels using the Proteome Profiler Mouse Cytokine Array Kit, Panel A (Catalog # ARY006, R&D Systems Inc., Minneapolis, MN). The membrane images were scanned and analyzed using ImageJ.

In addition, plasma TG levels were measured using the TG Colorimetric Assay Kit (EEA028, ThermoFisher Scientific) based on the introduction of the manufacturer.

### 2.11. Statistics

Data were analyzed using GraphPad Prism (Version 10.1.2) and expressed as mean ± SEM. Data normality was assessed using the D'Agostino–Pearson normality test to determine the appropriate use of either a 2-tailed Student *t*-test, Mann–Whitney test, or one- or two-way ANOVA for comparisons of two or more groups or factors. Dunnett's multiple comparisons test was used as the post hoc test for one-way ANOVA. A mixed-effects two-factor ANOVA was applied for comparisons across multiple time points and two factors. In the mixed effects model, genotype was treated as the random effect, and time as the fixed effect. A *p* value of ≤ 0.05 was considered statistically significant.

## 3. Results

### 3.1. TYMP Expression Is Increased in AAA Vessel Walls

**Figure 1:**
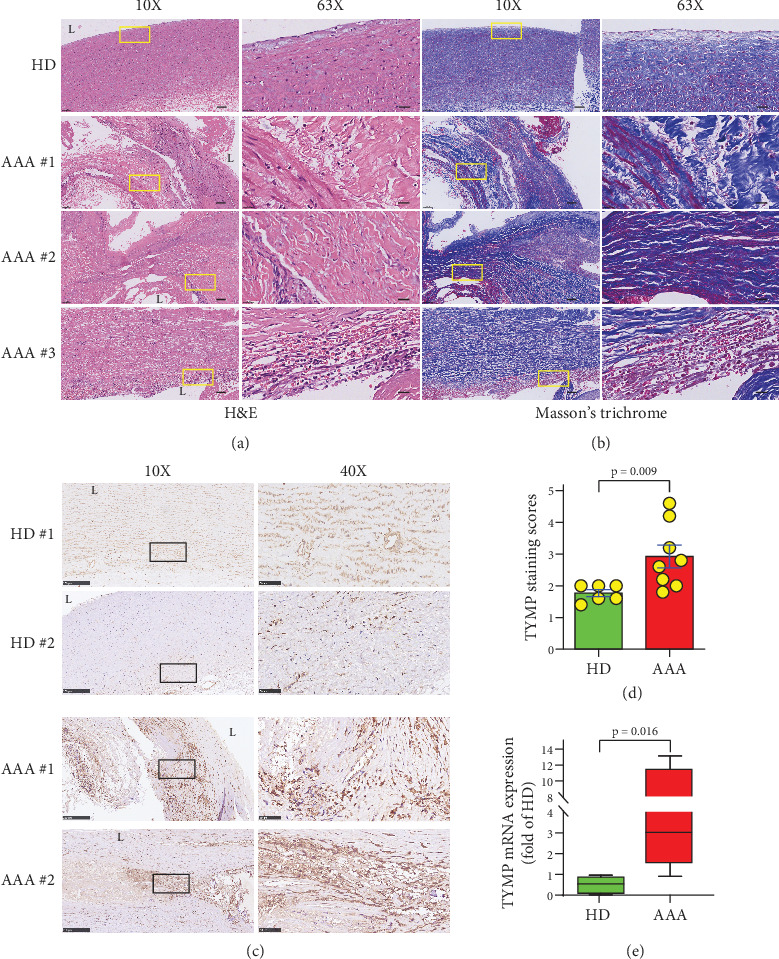
TYMP is elevated in the human abdominal aortic aneurysm vessel wall. Human AAA vessel walls and normal abdominal aorta from healthy donors (HD) were sectioned for analysis. (a) H&E staining. (b) Masson's trichrome staining. Scale bars: 100 *μ*m for 10× images and 20 *μ*m for 63× images. (c) Immunohistochemical staining for TYMP. Brown indicates a positive stain. Scale bars: 250 *μ*m for 10× images and 50 *μ*m for 40× images. (a–c) Samples from 6 HD and 8 AAA patients. Rectangular boxes in the 10× images indicate regions shown at higher magnification (63× or 40×). (d) TYMP staining scores. *N* = 6 for HD and *N* = 8 for AAA groups. The Student *t*-test was used. (e) qPCR analysis of TYMP expression in aortic vessel walls harvested from HD and AAA patients. *N* = 6 in HD and 8 in AAA groups. The Mann–Whitney test was used.

To assess the involvement of TYMP in AAA pathogenesis, we studied 16 AAA patients and presented their clinical data in Supporting Information 1: Table S1. Compared to the aortic walls of healthy donors, AAA patient samples showed significant structural disruption, as demonstrated by H&E and Masson's trichrome stains (Figures 1(a) and 1(b)). We observed a disorganized VSMC layer, accumulating blood cells, plaque, fat, and connective tissue. We also observed a pronounced increase in disoriented fibrotic tissue, compromising the structural integrity of the VSMC layer.

Consequently, we compared TYMP expression in normal aorta and AAA by IHC and qPCR. As shown in Figure 1(c), TYMP expression was detectable in both healthy aortas and AAA vessel walls; however, the staining intensity and score were significantly higher in the aneurysm samples (Figure 1(d)). The majority of the TYMP was expressed within the vasa vasorum in the healthy aorta. However, TYMP was expressed by all cells in the AAA vessel wall, with a significant increase observed in VSMCs (Supporting Information 3: Figure S3). qPCR analyses further confirmed the increase of TYMP mRNA in AAA samples compared to the healthy aortas (Figure 1(e) and Supporting Information 3: Figure S4). These data suggest that TYMP expression is increased in the AAA vessel wall compared to healthy controls.

### 3.2. TYMP Deficiency in Mice Reduces the Incidence of AAA

**Figure 2:**
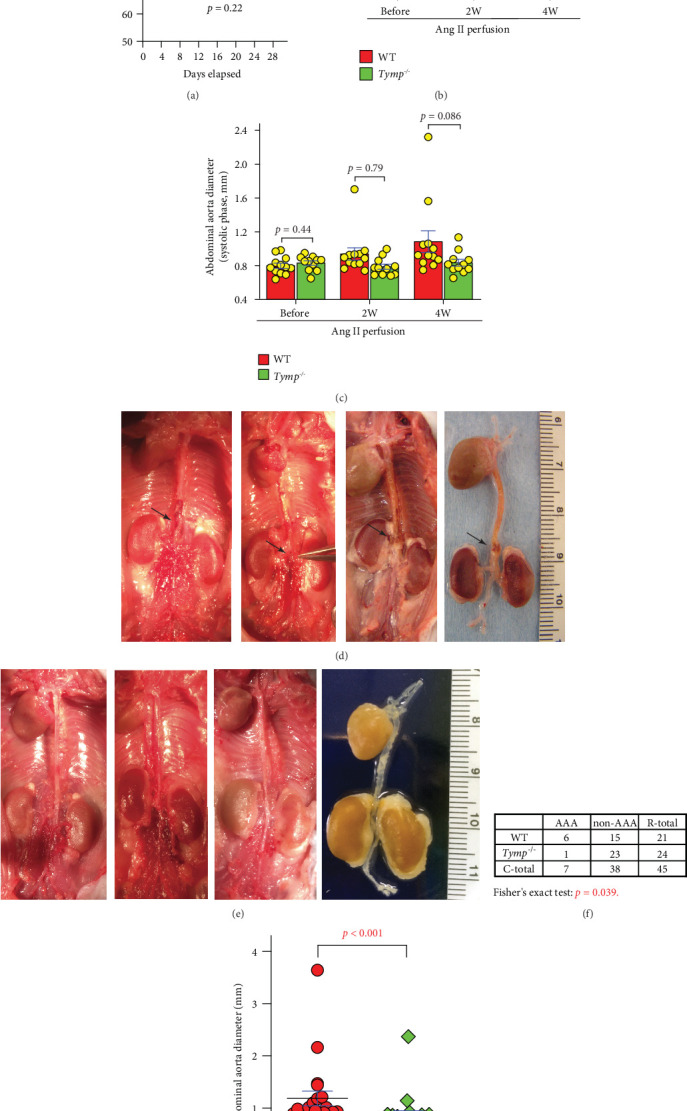
TYMP deficiency reduces the prevalence of AAA in mice. Wild-type (WT) (*n* = 26) and *Tymp^−/−^* (*n* = 26) mice were fed a Western diet starting at 4 weeks of age for 8 weeks. Mice then received chronic Ang II infusion in a dose of 1 *μ*g/kg/day delivered with an Alzet osmotic mini pump for 4 weeks. AAA development was monitored with echography. (a) Survival curve analysis (*n* = 26). Log-rank test was used. (b) Inner diameter of the abdominal aorta (AA) in the diastolic phase. (c) Inner diameter of AA in the systolic phase. (b, c) Data are shown as mean ± SEM (*n* = 12 for WT and 11 for *Tymp^−/−^*). The Student *t*-test was used for statistical analyses. (d) Gross finding of the aortic tree in WT mice. Arrows indicate AAA. (e) Gross finding of the aortic tree in *Tymp^−/−^* mice. (d, e) Scale: two continuous numbers on the ruler = 1 cm. (f) Contingency table showing the number of mice with or without AAA formation, as determined by necropsy. Fisher's exact test was used for the statistical analysis. (g) AA diameter at the suprarenal level measured by a caliper; *n* = 21 in WT and 24 in the *Tymp^−/−^* group. The Mann–Whiney test was used.

To investigate the role of TYMP in AAA development, we chronically perfused Ang II into WD-fed WT and *Tymp^−/−^* mice. *Tymp* deficiency did not significantly affect the body weight and plasma TG levels in these mice (Supporting Information 3: Figure S5). Five WT and two *Tymp*^*−*/*−*^ mice died prematurely, with the majority of these deaths occurring within 10 days after Ang II infusion. As illustrated in Figure 2(a), the *Tymp*^*−*/*−*^ group exhibited a lower, albeit statistically nonsignificant, mortality rate. Autopsies revealed hemorrhage in the thoracic cavity (Supporting Information 3: Figure S6). Heart rupture was not confirmed, suggesting an aortic rupture; however, due to advanced autolysis and decomposition of the carcasses, the bleeding site could not be determined. These mice were excluded from the subsequent analysis of AAA prevalence.

As shown in Figure 2(b) and Supporting Information 3: Figures S7A and S7C, infusion of Ang II for 4 weeks significantly increased the luminal diameter of the AA during the diastolic phase in WT mice, rising from an average of 0.925 ± 0.127 mm before Ang II administration to 1.25 ± 0.342 mm posttreatment (*p* = 0.006, *n* = 12). However, this effect was not observed in *Tymp^−/−^* mice, where the AA luminal diameter remained similar before and after Ang II infusion (0.904 ± 0.166 mm pre-Ang II vs. 0.984 ± 0.116 mm at 4 weeks, *p* = 0.2, *n* = 11). Consequently, the posttreatment diastolic luminal diameter in *Tymp*^*−*/*−*^ mice was significantly smaller compared to that in WT mice (Figure 2(b)). Additionally*^−^*while not reaching statistically significant, there was an observed trend towards increased AA luminal diameter in the systolic phase for WT mice, as depicted in Figure 2(c) and Supporting Information 3: Figures S7B and S7D, a trend not seen in *Tymp*^*−*/*−*^ mice.

Necropsy examination confirmed the presence of AAA in 6 out of 21 WT mice, resulting in an incidence rate of 28.6%, while in the *Tymp^−/−^* cohort, only 1 of 24 mice developed an AAA, representing a 4.2% incidence (Figures 2(d) and 2(e)). Fisher's exact test indicated that TYMP deficiency significantly reduced the prevalence of AAAs (Figure 2(f), *p* = 0.039). The identified aneurysms exhibited a fusiform shape, typically located at the suprarenal artery, with their longitudinal axis aligning with the artery. The average AA diameter measured above the renal artery was larger in WT mice compared to *Tymp*^*−*/*−*^ mice (Figure 2(g)). A receiver operating characteristic (ROC) analysis, using AA diameter in the *Tymp*^*−*/*−*^ group as the control and the WT group as the test group, indicated that the presence of TYMP is a significant risk factor for AA dilation (Supporting Information 3: Figure S8). The aneurysms appeared dark red, indicative of intramural thrombosis. It is important to note that although sudden deaths were attributed to hemorrhage in the thoracic cavity, no thoracic aortic aneurysms were confirmed in any surviving mice.

In addition, we evaluated the hemodynamics in the AA, including peak and mean velocities, as well as acceleration and deceleration patterns of blood flow before, 2-, and 4-week post-Ang II infusion. Data from Supporting Information 3: Figures S9A–S9D suggest that TYMP deficiency had no significant impact on these flow parameters at the evaluated time points. In contrast, among the three WT mice with confirmed AAAs, there was a significant increase in acceleration at 2- and 4-week postinfusion, as displayed in Supporting Information 3: Figure S9E (WT panel) and S9F.

### 3.3. TYMP Deficiency Attenuates the Distortion of the Aortic Wall in the Murine AAA Model

H&E staining of WT mouse AAA cross-section demonstrated dilated lumens (Supporting Information 3: Figure S10A). Within these aneurysms, we observed either freshly formed (WT#1) or fibrotic (WT#2) hematoma, which disrupted the media and adventitia of the affected aorta. In contrast, the aortic structure of *Tymp^−/−^* mice remained unchanged, preserving wall integrity even in the mouse with an aneurysm. Subsequent EVG staining highlighted that the elastic fibers within the vessel walls of *Tymp^−/−^* mice retained better integrity, contrasting with the breakage and disarray seen in the elastic fibers of the aortic wall of WT mice (Supporting Information 3: Figure S10B).

**Figure 3:**
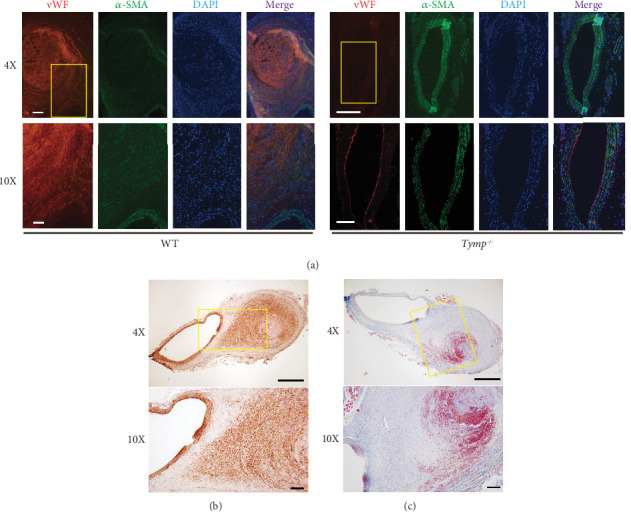
TYMP deficiency attenuates vessel wall structural distortion in the murine AAA model. (a) Paraffin-embedded sections of AAA vessel walls from WT mice and AA vessel walls from *Tymp^−/−^* mice were subjected to double immunofluorescence staining for vWF and *α*-SMA. Nuclei were stained with DAPI. Scale bars: 200 *μ*m for 4× images and 100 *μ*m for 10× images. (b) IHC for *α*-SMA in AAA sections from WT mice. Brown indicates positive staining. (c) Masson's trichrome staining of the AAA sections. (b, c) Scale bars: 1 mm for 4× images and 50 *μ*m for 10× images. Images of the WT group represent three AAA samples, while images in the *Tymp^−/−^* group represent six randomly selected AAs. Rectangular boxes in the 4× images indicate regions magnified in the 10× images.

Double immunofluorescence staining for von Willebrand factor (vWF), expressed in both endothelial cells and platelets (and thus platelet-rich thrombus), and *α*-SMA, a VSMC marker, showed disruption of the endothelial layer in the aortas with aneurysm (Figure 3(a)). The high amount of vWF in aneurysmal hematoma areas indicated platelet-rich thrombus formation. The aorta of *Tymp*^*−*/*−*^ mice maintained a normal structure across all layers, consistent with H&E and EVG staining results. The *α*-SMA positive signal found within the hematoma was verified through IHC (Figure 3(b)), and Masson's trichrome staining showed these regions as fibrotic (in blue) interwoven with blood cells (in red) (Figure 3(c)). This suggests that the *α*-SMA positive cells within the hematoma may be myofibroblasts involved in the pathological response to aneurysm formation or healing. No CD68-positive macrophage accumulation was observed in both WT and *Tymp^−/−^* vessels (data not shown).

### 3.4. TYMP Enhances MMP2 Production and Secretion in VSMCs

**Figure 4:**
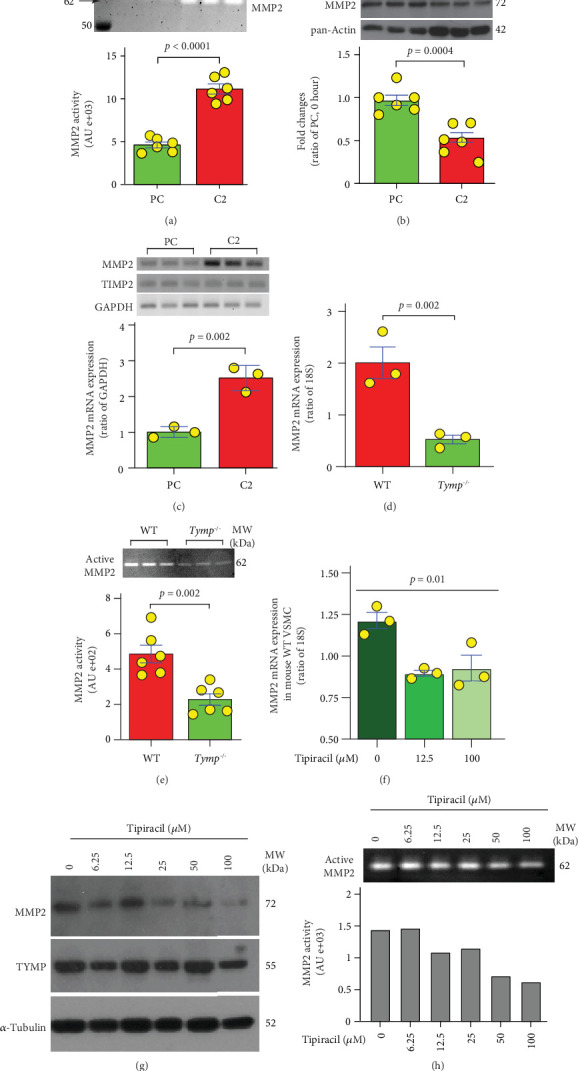
TYMP enhances MMP2 production and secretion in VSMCs. C2 and PC cells (1.00e + 06) were cultured in a 6-cm dish for 8 h, washed with warm PBS, and then incubated in serum-free DMEM for 24 h. (a) The media were collected and used for a gelatin zymography assay (*N* = 6). (b) Cells were harvested for Western blot assay of MMP2. Data are normalized to pan-actin and showed as fold change relative to PC (*N* = 6). (c) Part of the cells prepared above were used for RNA extraction, followed by an RT-PCR assay (*N* = 3). (d) WT and *Tymp^−/−^* VSMCs (5.00e + 05 cells/well) were seeded into a 6-well plate and cultured in full culture media for 24 h, and then, total RNAs were extracted for the qPCR assay (*N* = 3). (e) WT and *Tymp^−/−^* VSMCs (5.00e + 05 cells/well) were seeded into 6-well plates and cultured for 24 h. The cells were rinsed with PBS and then cultured in serum-free DMEM for another 24 h. The media were collected for zymography assay (*N* = 6). (a–e) The Student *t*-test was used for statistical analyses. (f) WT VSMCs (5.00e + 05 cells/well) were seeded into 6-well plates and cultured overnight. The cells were rinsed with PBS and then cultured in serum-free media in the presence or absence of tipiracil for another 24 h. Cells were collected for qPCR assay of MMP2 expression (*N* = 3). One-way ANOVA was used for the statistical analysis. (g, h) C2 cells (1.00e + 06/6-cm dish) were cultured in serum-free media in the presence of different concentrations of tipiracil for 24 h, and then, cells and media were collected for (g) Western blot and (h) zymography assay of MMP2 expression. Results are representative of two biological repeats.

MMPs, particularly MMP2 and MMP9, are known to play crucial roles in AAA formation [8, 25]. TYMP expression has been shown to correlate positively with MMP2 and MMP9 [16, 26]. Consequently, we measured MMP2/9 activity in C2 and PC cell–conditioned media by gelatin zymography and found that overexpressing TYMP significantly increased MMP2 activity (Figure 4(a)). MMP9 was undetectable under these conditions. However, intracellular levels of MMP2 were substantially reduced in C2 cells when assessed via Western blotting (Figure 4(b)). Therefore, we examined the expression of MMP2 at the mRNA levels and found it was significantly increased in C2 cells (Figure 4(c)), aligning with its changes in activity. These data indicate that TYMP promotes both MMP2 transcription and secretion in VSMCs.

These results were corroborated using VSMCs primarily cultured from WT and *Tymp*^*−*/*−*^ mice. As shown in Figures 4(d) and 4(e), TYMP deficiency markedly decreased MMP2 mRNA expression and its activity in these cells. Tipiracil treatment also dose-dependently reduced MMP2 expression at mRNA, protein, and activity levels (Figures 4(f), 4(g), and 4(h)). Taken together, these data suggest that TYMP plays an important role in enhancing MMP2 expression and secretion, which could contribute to the development and progression of AAA.

### 3.5. TYMP Enhances the Expression of Proinflammatory Cytokines

**Figure 5:**
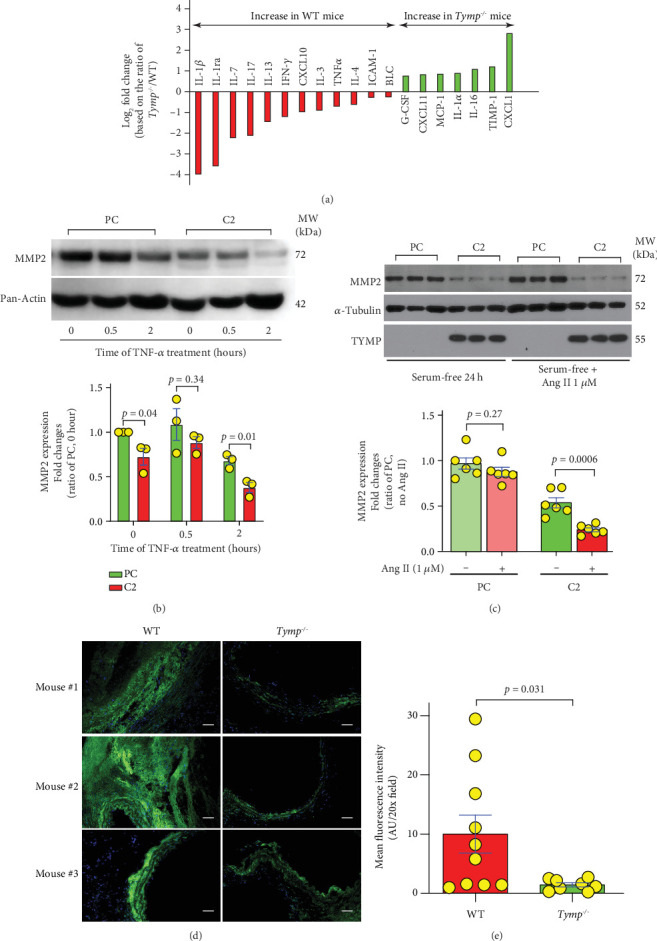
TYMP is systematically proinflammatory and enhances TNF-*α*- and Ang II-stimulated MMP2 production and secretion in VSMCs. (a) Plasma samples pooled from 6 WT mice (3 with AAA and 3 randomly selected) and 6 *Tymp^−/−^* mice (1 with AAA and 5 randomly selected) were analyzed using the Proteome Profiler Mouse Cytokine Array. The intensity of each spot was quantified using ImageJ. The ratio of *Tymp^−/−^* to WT was calculated, and the average log2 fold change for each protein (two spots per protein) is displayed. Molecules with changes greater than 25% were shown. (b) C2 and PC cells (1.00e + 06/6-cm dish) were serum-starved overnight and then treated with TNF-*α* (10 ng/mL) in serum-free media for the indicated durations. Cells were harvested for Western blot analysis of MMP2 expression (*N* = 3). (c) C2 and PC cells (1.00e + 06/6-cm dish) were serum-starved for 24 h, treated with 1 *μ*M Ang II in serum-free DMEM for 24 h, and then harvested for Western blot assay (*N* = 6). (d, e) Three AAA from the WT mice and one AAA (#3) and two randomly selected AA (#1 and 2) from *Tymp^−/−^* mice were embedded in OCT and sectioned into 6-*μ*m slices. Three slides from each mouse, carrying 3–4 sections, were used for the DQ gelatin-based in situ zymography assay of MMP activity. (d) Representative images of MMP activity in vessel walls. Scale bar = 50 *μ*m. (e) Mean fluorescence intensity, representing MMP activity in the vessel wall, was analyzed by ImageJ. *N* = 10 in WT and 8 in *Tymp^−/−^* mice. (b, c, e) The Student *t*-tests were used for statistical analyses.

Proinflammatory cytokines and chemokines, including TNF-*α* [27], interleukin (IL)-1*β* [28], IL-17 [29], and IL-23 [30], have been implicated in AAA formation and disease progression. We recently demonstrated that TYMP expression is increased in COVID-19 patients and its expression is significantly correlated with COVID-19-associated inflammation and thrombosis [31]. To determine TYMP's role in promoting inflammation and subsequent AAA development, we performed a Proteome Profiler Mouse Cytokine Array on plasma pooled from six WT and six *Tymp*^*−*/*−*^ mice. The analysis, as depicted in Figure 5(a) and Supporting Information 3: Figure S11, revealed elevated levels of various cytokines and chemokines in WT plasma, many of which are established contributors to AAA formation. Interestingly, the levels of IL-1*α* and the tissue inhibitor of metalloproteinases 1 (TIMP1) were found to be significantly lower in the WT mice plasma. The suppression or genetic elimination of IL-1*α* or TIMP1 has also been associated with the progression of AAA [32, 33].

To investigate the relationship among TYMP, inflammation, and MMPs, we treated serum-deprived C2 and PC cells with TNF-*α* and assessed MMP2/9 expression and activation. As shown in Figure 5(b), baseline intracellular MMP2 levels were naturally lower in C2 cells. Following TNF-*α* exposure for 30 min, a slight uptick in MMP2 expression was observed, followed by a substantial decrease at the 2-h mark in both cell types, indicating increased MMP2 secretion. Two hours post-TNF-*α* treatment, MMP2 activity became detectable in C2 cells (albeit undetectable in PC cells), as evidenced by a very faint signal on zymography (Supporting Information 3: Figure S12A with a yellow arrow). This implies that TNF-*α* stimulates MMP2 secretion, which is further augmented by TYMP.

Additionally, to probe the combined effects of TYMP and Ang II on MMP2 expression, we administered 1 *μ*M Ang II to serum-free PC and C2 cells over 24 h. According to Figure 5(c), Ang II had no significant impact on MMP2 levels within PC cells. However, in C2 cells, Ang II treatment moderately but significantly reduced intracellular MMP2 levels, suggesting an increase in MMP2 secretion. This effect, however, was not verified by zymography (Supporting Information 3: Figure S12B).

In vivo examination through in situ zymography of aortic sections from WT mice with confirmed AAAs and from *Tymp*^*−*/*−*^ mice, specifically one with AAA and two without, corroborated the in vitro findings. TYMP deficiency was shown to substantially decrease MMP activity in the aortic walls, as displayed in Figures 5(d) and 5(e), further supporting the association between TYMP, inflammation, and AAA development.

### 3.6. TYMP Enhances AKT Phosphorylation in VSMCs

**Figure 6:**
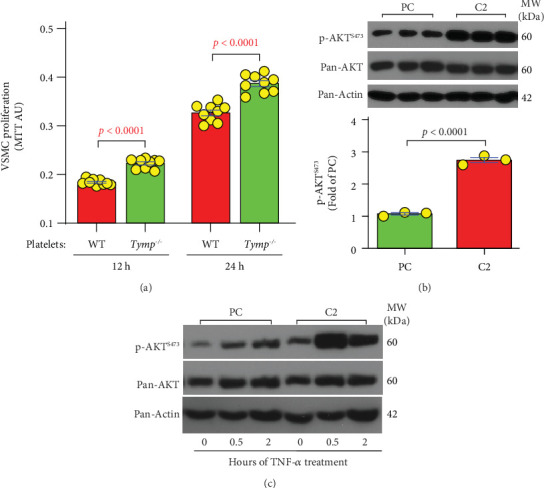
TYMP-expressing platelets inhibit VSMC proliferation, and TYMP overexpression leads to constitutive AKT activation in VSMCs. (a) Murine WT VSMCs (1.00e + 03/well) were seeded into 96-well plates, serum-starved overnight, and then stimulated with FCM containing WT or *Tymp^−/−^* platelets (1.00e + 07 platelets/well). Cell proliferation was measured after 12 and 24 h using an MTT assay (*N* = 9). (b) C2 and PC cells (1.00e + 06/6-cm dish) were cultured overnight in FCM, and AKT activation and expression were analyzed via Western blot (*N* = 3). (a, b) The Student *t*-test was used for statistical analyses. (c) C2 and PC cell lysates prepared as described in Figure 5(b) were used for Western blot assay of AKT activation. This panel represents two biological repeats. Note: the pan-actin loading control is shared with Figure 5(b).

The presence of intraluminal thrombus is a regular occurrence in AAAs, but its effect on AAA enlargement remains incompletely understood. Platelets are known to contain high levels of TYMP [11, 34]. To investigate the direct impact of platelets on VSMC activity, we cocultured WT VSMCs with both WT and *Tymp^−/−^* platelets, which were washed to remove plasma components. As indicated in Figure 6(a), WT VSMCs treated with *Tymp^−/−^* platelets showed a higher proliferation rate than cells treated with WT platelets. These data suggest that TYMP, whether originating from platelets or other cellular sources, can impact VSMC functionality and potentially contribute to vessel wall weakening in certain pathological conditions.

The mechanism by which TYMP modulates MMP2 expression remains unclear. Our previous research involving platelets and mesenchymal stem cells hinted at a multifaceted interaction between TYMP and the AKT signaling pathway [10, 11]. We found that ADP-stimulated AKT phosphorylation was slightly higher in the early phase but was dramatically decreased in the late-phase activation of *Tymp^−/−^* platelets [10]. Conversely, TYMP-deficient mesenchymal stem cells exhibited continuous AKT phosphorylation, even though MMP2 activity was reduced in these cells [26]. These findings imply a dual role for TYMP in AKT pathway regulation: It may naturally inhibit AKT activity, but under certain conditions, it enhances AKT activation via an alternate pathway. As shown in Figure 6(b), we found that overexpression of TYMP in VSMCs significantly increased AKT phosphorylation at S473. TNF-*α* stimulated AKT activation in both cell types, with a more pronounced effect in C2 cells (Figure 6(c) and Supporting Information 3: Figure S13). Given the role of AKT activation in promoting MMP2 production in VSMCs and its significance in AAA development [35], these data suggest that TYMP-enhanced MMP expression could be mediated by the AKT pathway. This hypothesis is further supported by examining AKT activation in human AAA samples, which showed strong p-AKT staining, indicating an increase in AKT activity compared to healthy controls (Supporting Information 3: Figure S14).

### 3.7. TYMP Is Involved in Noncanonical TGF*β*1 Signaling, Increasing AKT Activation and Promoting the Expression of Connective Tissue Growth Factor (CTGF) and MMP Activity in VSMCs

**Figure 7:**
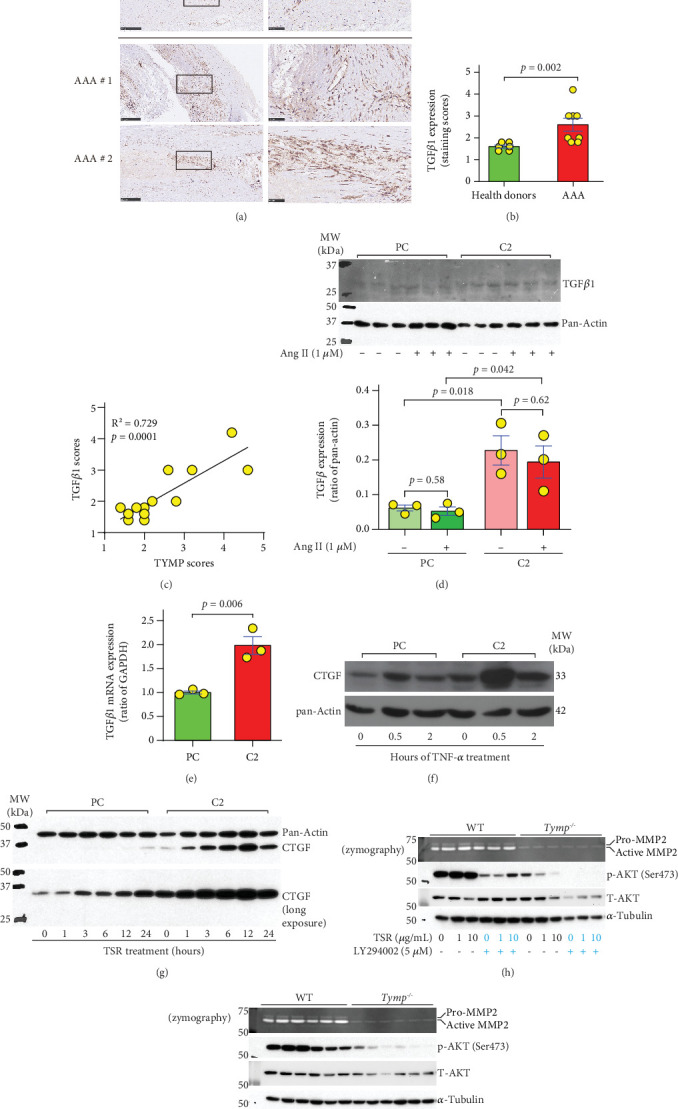
TYMP correlates TGF*β*1 expression in human AAA and enhances noncanonical TGF*β*1 signaling activation in VSMCs. (a) IHC of TGF*β*1 in human AAA vessel walls. Scale bar = 250 *μ*m for 10× images and 50 *μ*m for 40× images. Rectangular boxes in the 10× images indicate regions magnified in the 40× images. (b) TGF*β*1 staining scores in healthy donor (HD) AAs (*n* = 6) and AAA vessel walls (*n* = 8). (c) Correlation analysis between TYMP and TGF*β*1 expression in human AAA samples. XY pairs = 14. The Pearson correlation coefficient (*r*) was used. (d) Serum-starved PC and C2 cells (1.00e + 06/6-cm dish) were treated with serum-free DMEM with or without 1 *μ*M Ang II for 24 h. TGF*β*1 expression was assessed by Western blot, and band intensity was analyzed using ImageJ (*N* = 3). (e) PC and C2 cells (1.00e + 06/6-cm dish) were cultured under normal conditions for 24 h. RNA was extracted, and TGF*β*1 mRNA expression was examined by qPCR (*N* = 3). (f) Cell lysates from Figure 5(b) were used to examine CTGF expression. Data represent two biological repeats. (g) PC and C2 cells (1.00e + 06/6-cm dish) were serum-starved overnight and then treated with fresh serum-free media containing 10 *μ*g/mL TSR for the indicated durations. Cells were harvested for Western blot analysis of CTGF expression. Pan-actin served as the loading control. Data represent two biological repeats. (h, i) WT and *Tymp^−/−^* VSMCs (5.00e + 05/6-cm dish) were serum-starved overnight and then treated with fresh serum-free media containing TSR at indicated concentrations in the presence vehicle, LY294002, or SB431542 for 12 h. Media were collected for zymography assay, and cells were harvested for Western blot analysis of AKT and *α*-tubulin. (b, d, e) The Student *t*-test was used for statistical analyses.

MMP2 activates TGF*β*1 signaling and plays an important role in arterial aging, a risk factor for developing AAA. TGF*β*1 expression has been significantly increased in the plasma of AAA patients [36] or patients with aortic dilatation [37]. However, the role of TGF*β*1 in AAA development in mice is controversial, with studies reporting both beneficial [38] and detrimental [39] effects. To elucidate the involvement of TGF*β*1 in AAA pathogenesis, we analyzed its expression in human AAA tissues. As demonstrated in Figures 7(a) and 7(b), and Supporting Information 3: Figures S15 and S16, our studies revealed a significant upregulation of TGF*β*1 within the AAA vessel walls. Notably, TGF*β*1 levels exhibited a positive association with TYMP expression (Figure 7(c)), particularly in VSMCs (Supporting Information 3: Figure S16).

To further investigate the regulatory role of TYMP on TGF*β*1 expression in VSMCs, we assessed its expression under both baseline and Ang II-stimulated conditions. While Ang II had no discernible impact on TGF*β*1 levels in both PC and C2 cells, a marked increase in expression was observed in C2 cells (Figure 7(d)). This elevation in TGF*β*1 expression in C2 cells was further validated by qPCR analysis (Figure 7(e)). These findings suggest that TYMP may play a crucial role in enhancing TGF*β*1 expression in VSMCs, thereby potentially contributing to AAA development.

CTGF is a key mediator of tissue remodeling and fibrosis through its interactions with TGF*β*1 and integrin *α*v*β*3. The upregulation of CTGF is associated with the pathogenesis and progression of ascending thoracic aortic aneurysm [40]. Activation of integrin *α*v*β*3 plays an important role in the TYMP-mediated proangiogenic process [41]. We thus investigated whether CTGF mediates TYMP's effect in response to stimulation of TNF-*α* and TSR, which activates the TGF*β*1 signaling pathway. As depicted in Figure 7(f), TNF-*α* induced a greater increase in CTGF expression in C2 cells compared to that in PC cells. Similarly, TSR stimulated CTGF expression in a time-dependent manner in both cell lines, with C2 cells showing a significantly higher response (Figure 7(g)). This evidence supports the premise that TYMP is integral to TGF*β*1 signaling and influences CTGF expression.

To explore the correlation among TYMP, AKT, TGF*β*1, and MMP2, serum-starved WT and *Tymp^−/−^* VSMCs were pretreated with LY294002, a potent PI3K (a well-known upstream regulator of AKT) inhibitor, SB431542, a selective TGF*β*1 inhibitor, or vehicle in serum-free media for 1 h. The cells were then stimulated with different doses of TSR, without removing the vehicle, LY294002, or SB431542 from the media, for an additional 12 h. Media and cells were subsequently harvested for MMP2 activity assays and to assess the phosphorylation of AKT, Smad2, and Smad3. As shown in Figures 7(h) and 7(i), basal levels of p-AKT were significantly higher in WT VSMCs than in *Tymp^−/−^* cells. While TSR dose-dependently increased p-AKT in WT VSMCs, unexpectedly, it decreased AKT phosphorylation in *Tymp^−/−^* VSMCs. No Smad2/3 phosphorylation was detected under any conditions (data not shown). AKT phosphorylation was significantly attenuated by LY294002 in both WT and *Tymp^−/−^* VSMCs (Figure 7(h)). However, high-dose TSR still induced AKT phosphorylation in WT cells in the presence of LY294002, suggesting a PI3K-independent AKT activation. TSR-induced AKT activation in WT VSMCs was significantly inhibited by SB431542 (Figure 7(i)). Interestingly, TSR-increased MMP2 activity in WT VSMCs aligned with the changes of p-AKT, indicating a p-AKT-dependent effect. In contrast, while TSR inhibited AKT phosphorylation in *Tymp^−/−^* VSMCs, MMP2 activity showed indiscernible changes in response to either LY294002 or SB431542 (Figures 7(h) and 7(i)).

## 4. Discussion

In this study, we utilized a chronic Ang II infusion model in WT and *Tymp^−/−^* mice on a WD and demonstrated that TYMP plays a pivotal role in AAA development. Our findings revealed an increase of TYMP expression within human AAA vessel walls, and its deficiency in mice significantly reduced Ang II-induced aortic dilation and AAA incidence. Beyond the known inhibitory effect of TYMP in VSMCs [12–14], we identified additional key contributions of TYMP to AAA prevalence. Specifically, TYMP deficiency markedly altered the expression of inflammatory cytokines and chemokines linked to AAA development. Furthermore, TYMP not only boosts MMP2 production but also its secretion. TYMP triggers constant AKT phosphorylation when overexpressed in VSMCs, engages in the TSP1/TGF*β*1 pathway-mediated AKT activation and MMP2 production, and elevates TGF*β*1 and CTGF levels. This study is the first to highlight TYMP's essential role in creating a conducive environment for AAA development, a major human vascular disease.

Human AAA formation spans years or even decades, influenced by a complex interplay of pathophysiological elements and risk factors, creating a highly intricate scenario. The prevalence of AAA, depending on geography, can be as high as 8% in men over the age of 65 [42, 43]. The ideal animal model for studying AAA should closely mimic both the pathophysiological processes and the characteristics of human AAA [44]. Especially, there is no ideal model to study AAA risk in children with obesity beginning in childhood. Starting the mice on a WD at 4 weeks of age mimics pediatric obesity resulting from early-life exposure to a high-fat, high-sugar diet. This early initiation allows us to observe the effects of prolonged metabolic stress and inflammatory responses on the vasculature, potentially contributing to earlier AAA onset. We chose an 8-week duration of WD feeding following by adding Ang II perfusion for another 4 weeks based on studies indicating that extended WD feeding in mice results in physiological changes consistent with human obesity and vascular abnormalities, which are critical for observing measurable AAA formation [5, 6, 45].

Extensive research has explored the impact of genetic factors on AAA development using *Apoe^−/−^* or *Ldlr^−/−^* mice, where the prevalence of AAA can reach 50-100% [46–48]. Studies have shown that *Apoe* gene products protect against oxidative damage [49], and *Apoe* gene deficiency alters the expression of 182 proteins [50]. Therefore, while introducing gene modifications into the *Apoe^−/−^* or *Ldlr^−/−^* background increases AAA prevalence and provides valuable insights into aortopathy etiology, these models may not closely replicate human AAA pathophysiology. This discrepancy arises because *APOE* or *LDLR* deficiencies are rare in humans, and double gene deficiencies are almost nonexistent. Using models with fewer genetic modifications may offer fresh perspectives more aligned with the pathophysiology of human AAA, particularly when testing the function of a single gene. In our study, after chronic Ang II infusion in mice fed WD, we observed a 28.6% incidence of AAA in WT mice. This rate surpasses that of a prior study, where only 20% of C57BL6 mice fed a standard laboratory diet developed AAA [17]. The pathological alterations, morphology, characteristics of AAA, and morbidity in WT mice closely resembled those seen in human AAA, indicating that this model effectively replicates the pathophysiology of AAA and is suitable for studying AAA. The diet protocol also mirrors the lifestyle prevalent in the Western world, particularly in the United States, suggesting that obese children could represent a cohort at high risk for developing AAA in the future.

Our model's higher AAA incidence indicates that systemic changes associated with the WD promote AAA development. Our previous and ongoing studies in a different project showed that feeding mice with WD dramatically enhanced thrombosis [10] and increased TYMP expression in platelets and livers (data not shown), respectively. While WD is well known to cause systemic chronic inflammation [7], this study is the first to demonstrate that TYMP plays an important role in WD-induced systemic inflammation. This aligns with several studies indicating TYMP's role in upregulating inflammatory cytokines like IL-8 and CXCL10 [9] and its positive correlation with plasma C-reactive protein levels, a common clinical inflammation marker [31]. These novel findings about TYMP-mediated alterations of inflammatory cytokines highlight TYMP's involvement in AAA pathology. However, additional studies are necessary to elaborate on how TYMP influences the expression of these inflammatory cytokines, especially concurrently.

The exact pathophysiology behind aneurysm formation remains unclear. However, it is widely recognized that VSMCs are the predominant cell type involved and play crucial roles in this process. Dysregulated VSMC function, behavior, and antioxidant status have been linked to vascular diseases, including neointimal hyperplasia, atherosclerosis, and AAA [51]. Studies from our laboratory and others have demonstrated that TYMP plays an inhibitory role in regulating VSMC function [12–15, 52]. In this study, we further demonstrated that VSMC treated with WT platelets showed a lower proliferation rate than cells treated with *Tymp^−/−^* platelets. Recent studies have demonstrated that the accelerated growth of AAA is associated with platelet activation and thrombosis in aneurysmal segments [53]. Thus, we predict that besides TYMP's contribution to promoting inflammation, its role in augmenting platelet activation and thrombosis, along with its inhibitory impact on VSMC functionality, collectively creates a conducive environment for the development of AAA.

In a canine transmyocardial laser revascularization model, we showed that laser treatment-enhanced angiogenesis is correlated with the increased expression of TYMP, MMP2, MMP9, and urokinase-type plasminogen activator (uPA) [16, 22], suggesting a potential association between TYMP, MMP2/9, and uPA. The activity of MMPs is strictly controlled at several levels, including transcription, production of zymogen (72 kDa), and activation (62 kDa), as well as binding to their natural endogenous inhibitors, TIMPs. While TYMP overexpression does not alter TIMP2 levels (Supporting Information 3: Figure S17), we observed a reduction in TIMP1 levels in WT plasma, as depicted in Figure 5(a). This observation aligns with in situ zymography results, which demonstrated increased MMP activity in the aortic walls of WT mice.

Various aortopathy has been linked to the gain-of-function mutation or dysregulation of the TGF*β*1 signaling pathway [54]. As mentioned above, the role of TGF*β*1 in the development of AAA is controversial, and both beneficial and detrimental effects have been reported [38, 39]. A recent review further highlighted that both activation and inhibition of TGF*β*1 disrupt its vital role in maintaining normal vascular biology, potentially leading to aneurysm formation by triggering both canonical and noncanonical signaling pathways [54]. Interestingly, the AKT pathway, a nonclassical pathway downstream of TGF*β*1, is implicated in the development of aortic aneurysms [35]. TGF*β*1 is secreted in a latent form by cells, binding to latent TGF*β*1 binding protein and the TGF*β*1 propeptide (also known as latency-associated peptide). We found that TYMP not only enhances TGF*β*1 transcription but also increases the active form of TGF*β*1 in VSMC, confirmed by an increase of the 25 kDa TGF*β*1. These in vitro studies reinforce the observed positive correlation between TYMP and TGF*β*1 expression in human AAA tissues, suggesting that TYMP-enhanced TGF*β*1 in VSMCs may have a harmful impact and enhance AAA development. However, TYMP does not exhibit a synergistic effect with Ang II in regulating MMP2 production, activation, or TGF*β*1 expression. We observed that Ang II does not influence MMP2 production and activity in VSMCs. This finding is consistent with several existing studies that found that Ang II did not affect constitutively expressed MMP2 in VSMCs and cardiac fibroblasts [55, 56].

In addition to MMP2, uPA is also known to activate TGF*β*1. uPA-mediated TGF*β*1 activation requires the binding of CD36 and TSP1 [57], a matricellular protein that plays an important role in cell–cell and cell–matrix interaction [23]. Similar to the TGF*β*1, although controversial, both salutary [58] and detrimental [59] effects of TSP1 have been reported in the AAA milieu. We have extensively studied the role of CD36 in the development of cardiovascular disease and recently found that TSP1-TSR/CD36 signaling enhances VSMC proliferation [60, 61]. CD36 deficiency did not reduce CTGF expression in VSMCs (data not shown), suggesting that TSP1/CD36 signaling does not affect CTGF. Therefore, TSR-induced, TYMP-facilitated increase of CTGF expression, AKT phosphorylation, and MMP activity is most likely through the activation of the TGF*β*1 signaling pathway [23], which was validated with the use of TGF*β*1 pathway inhibitor, SB431542. CTGF participates in diverse biological processes, including growth and development; however, overexpression of CTGF is correlated with severe fibrotic disorders and a proinflammatory status in VSMCs that leads to endothelial dysfunction [62, 63]. Through multiple positive feedback loops, CTGF could enhance TGF*β*1 signaling [62], further leading to an unbalanced homeostasis of the VSMC environment, which may contribute to the development of AAA. Additional studies are needed to clarify this speculation.

## 5. Conclusion

Our research revealed that TYMP, an enzyme in the pyrimidine salvage pathway, possesses multifaceted functions, particularly in modulating vascular biology. It plays a significant role in the function of VSMCs and systemic inflammation. TYMP-mediated changes in the microenvironment may contribute to the progression of AAA. This study introduces a novel mechanistic target for AAA treatment. Further investigation is needed to explore the clinical applicability of this discovery, particularly using the TYMP-selective inhibitor, tipiracil, an FDA-approved drug that has already been shown to inhibit thrombosis in mice.

## Data Availability

All data supporting this paper's findings have been involved in the paper, and data marked as “data not shown” are available from the corresponding authors upon reasonable request.

## Nomenclature

*α*-SMA: *α*-smooth muscle actin
AA: abdominal aorta
AAA: abdominal aortic aneurysm
Ang II: angiotensin II
CTGF: connective tissue growth factor
EVG: Elastica van Gieson
FCM: full culture media
IVC: inferior vena cava
MMP: matrix metalloproteinase
Smad: suppressor of mothers against decapentaplegic
TGF*β*1: transforming growth factor beta1
TIMP: tissue inhibitor of metalloproteinase
TNF-*α*: tumor necrosis factor-alpha
TSP1: thrombospondin-1
TSR: thrombospondin-1 type 1 repeat domain
TYMP: thymidine phosphorylase
uPA: urokinase-type plasminogen activator
VSMC: vascular smooth muscle cells
WD: Western diet

## Supporting Information

Supporting Information 1 Table S1. AAA patient information.

Supporting Information 2 Table S2. Primers for testing the targeted genes by real-time PCR.

Supporting Information 3 Figure S1. Flow chart of the proposed study. Figure S2. Histochemical staining images were scored based on staining intensity. We adopted a 6-point scale system, and representative images with score assignments are provided. Scale bar = 250 *μ*m. Figure S3. TYMP expression is increased in the human AAA vessel wall. The human AAA vessel wall and healthy control aorta vessel wall (Con) were sectioned and double stained for TYMP and *α*-SMA. Nuclei were stained with DAPI. Scale bar = 200 *μ*m. Figure S4. Melting curve analysis was performed to confirm the amplicons of human thymidine phosphorylase (TYMP) and glyceraldehyde 3-phosphate dehydrogenase (GAPDH). Figure S5. *A*. WT and *Tymp^−/−^* mice were fed a Western diet starting at 4 weeks of age for 8 weeks. Mouse body weight was monitored weekly until the implantation of the Alzet osmotic mini pump. *B*. Whole blood was drawn via inferior vena cava puncture at the time of sacrifice, and plasma was isolated by centrifugation using 0.109 M sodium citrate as an anticoagulant (1:9). Plasma triglyceride levels were measured using the triglyceride (TG) Colorimetric Assay Kit (EEA028, ThermoFisher Scientific). Figure S6. Necropsy of mice died after Ang II perfusion. Figure S7. Thymidine phosphorylase (TYMP) deficiency reduced the prevalence of abdominal aortic aneurysm (AAA) formation in mice. Mice were treated as mentioned in Figure 2. *A* Inner diameter of the abdominal aorta in the diastolic phase. *B* Inner diameter of the abdominal aorta in the systolic phase. Data are shown as mean ± SEM. *N* = 12 in WT and 11 in *Tymp^−/−^* groups for both A and B. One-way ANOVA was used to determine the time-dependent changes. *C* Representative echo images in the diastolic phase. *D* Representative echo images in the systolic phase. Figure S8. A receiver operating characteristic (ROC) analysis using the data from Figure 2(g) (abdominal aorta diameter), with the *Tymp^−/−^* group as the control and the WT group as the test group. Figure S9. Thymidine phosphorylase (TYMP) deficiency reduced the prevalence of abdominal aortic aneurysm (AAA) formation in mice. Mice were treated as mentioned in Figure 2. *A* Peak velocity, *B* mean velocity, *C* acceleration, and *D* deceleration were determined. Two-way ANOVA was used to analyze the interaction between genotype and times in Panels A–D. *E* Representative acceleration echo images in the WT and *Tymp^−/−^* mice. *F* Statistical analysis of acceleration in the three WT mice with AAA. One-way ANOVA was used for the statistical analyses for all panels. Data are shown as mean ± SEM. *N* = 12 in WT and 11 in *Tymp^−/−^* groups for Panels A–D. Panel E represents three mice. *N* = 3 in Panel F. Figure S10. TYMP deficiency attenuates distortion of vessel wall structure in the mouse AAA model. *A* H&E staining. *B* Elastica van Gieson (EVG) staining. ∗ indicates vessel true lumen. # indicates hematoma. Images of the WT group represent three AAA samples, and images of the *Tymp^−/−^* group represent six randomly selected AAs. Figure S11. Expression of plasma cytokines in WT and *Tymp^−/−^* mice fed a Western diet and underwent Ang II treatment. Plasma pooled from six WT mice (three with AAA and three randomly selected) and six *Tymp^−/−^* mice (one with AAA and five randomly selected) were subjected to the Proteome Profiler Mouse Cytokine Array, Panel A. Figure S12. TYMP enhances MMP2 production and secretion in VSMCs. *A* C2 and PC cells (1.00e + 06) were seeded into a 6-cm dish and cultured for 8 h under normal conditions. Cells were synchronized by serum starvation overnight and then treated with TNF-*α* (10 ng/mL) for the indicated durations. Both the overnight-conditioned media and media after TNF-*α* treatment were subjected to zymography. The yellow arrow indicates the weak MMP2 band. Data represent two biological repeats. *B* PC and C2 cells (1.00e + 06) were serum-starved for 24 h and then treated with Ang II at a final concentration of 1 *μ*M in serum-free DMEM for an additional 24 h. The culture media were collected and used for gelatin zymography. Data are presented as fold changes of average PC before Ang II treatment. Data are shown as mean ± SEM. *N* = 6 in the Student *t*-test was used. Figure S13. TYMP overexpression enhances AKT signaling activation in VSMCs. Serum starvation–synchronized C2 and PC cells (1.00e + 06) were treated with TNF-*α* for the indicated durations, and cell lysates were used for Western blot assay of AKT activation as shown in Figure 6(c). Band intensity was analyzed using ImageJ, and p-AKT expression was adjusted with the expression of total AKT. Data were shown as fold change of PC at 0 h. The graph shows data of two biological repeats. Figure S14. Evaluation of AKT activation in healthy human aortic vessel wall and AAA samples. Paraffin-embedded human aortic tissues were sent to Bios Biological (https://biossci.com/), a professional company for histological study. Immunohistochemical staining of p-AKT was conducted using an antibody against phospho-pan-AKT1/2/3 (Ser473) (AF0016, Affinity), which detects endogenous levels of pan-AKT1/2/3 only when phosphorylated at Sersine 473. Positive staining is indicated by brown coloration, while nuclei were stained with hematoxylin (blue). The images were scanned and scored by lay individuals based on staining intensity showed in Figure S1, and score data were used for statistical analysis, with results presented in bar graph format. Data are shown as mean ± SEM. *N* = 6 in the healthy donor (HD) group and 8 in the AAA group. The Student *t*-test was used. Scale bar = 250 *μ*m in 10× images and 50 *μ*m in 40× images. Figure S15. IHC of TGF*β*1 in human healthy donor (HD) aorta or in the AAA vessel walls. Brown indicates positive staining. Scale bar = 250 *μ*m in 10× images and 50 *μ*m in 40× images. Figure S16. TGF*β*1 expression is increased in the human AAA vessel wall. The human AAA vessel wall and healthy control aorta vessel wall (Con) were sectioned and double stained for TGF*β*1 and *α*-SMA. Nuclei were stained with DAPI. Scale bar = 200 *μ*m. Figure S17. TYMP overexpression does not affect TIMP2 expression in VSMCs. As mentioned in Figure 7(g), serum-starved PC and C2 cells (1.00e + 06) were treated with serum-free media in the presence of 10 *μ*g/mL TSR for the indicated durations. TIMP2 levels were examined by Western blot, with *β*-tubulin blotted as the loading control.
