# Supplementary material for: Thymidine Phosphorylase Promotes Abdominal Aortic Aneurysm via VSMC Modulation and Matrix Remodeling in Mice and Humans
Source: Cardiovasc Ther. 2024 Dec 18;2024:1129181. doi: 10.1155/cdr/1129181 (PMC11669429; doi:10.1155/cdr/1129181)
Supplement: Supporting Information 1 — Table S1. AAA patient information. [file 1129181.f1.pdf]

Supplementary Table 1. AAA patient information.

AAA Patient ID	Sex	Age	Race	Primary Diagnosis	Secondary Diagnosis	Complications	AAA diameter (mm)
#1	M	70	Asian	AA Dissection	arteriosclerosis	T2DM, hypertension	53
#2	M	56	Asian	AAA	Arteriosclerosis Obliterans of Lower Extremities	Hyperuricemia, gout, hyperlipoidemia, calculus of gallbladder	83
#3	F	67	Asian	AAA	coronary atherosclerosis	Hypertension, intervertebral disc herniation	61
#4	M	66	Asian	AAA	abdominal arteriosclerosis	Hypertension	50
#5	M	58	Asian	AAA	abdominal arteriosclerosis	Diffuse large B cell lymphoma (DLBCL), bladder stone, hyperplasia of prostate, syphilis	44
#6	M	73	Asian	AAA	coronary atherosclerosis	Hypertension	95
#7	F	85	Asian	AAA	coronary atherosclerosis	Anemia, kidney stone, chest effusion	110
#8	M	71	Asian	AAA	Abdominal atherosclerosis	Hypertension	80
#9	M	84	White	AAA	aneurysm wall atherosclerosis	Kidney disease, Parkinson’s	information unavailable
#10	F	91	White	AAA	abdominal aorta-aneurysm repair. changes with aneurysm wall atherosclerosis	Neck and lumbar surgery, pacemaker/defibrillator	information unavailable

#11	F	75	White	AAA	atherosclerosis consistent with aneurysm	VP shunt, hydrocephalus	information unavailable
#12	M	information unavailable	White	AAA	plaque; aneurysm repair; atherosclerotic plaque	information unavailable	information unavailable
#13	M	69	White	AAA	complicated atherosclerosis	emphysema/COPD, pacemaker	information unavailable
#14	M	77	White	AAA	consistent with dissecting aneurysm	information unavailable	information unavailable
#15	M	87	White	AAA	focal cystic medial degeneration	Arthritis, degenerative disk disease	information unavailable
#16	M	information unavailable	White	AAA	calcification	information unavailable	information unavailable
