# Supplementary material for: Thymidine Phosphorylase Promotes Abdominal Aortic Aneurysm via VSMC Modulation and Matrix Remodeling in Mice and Humans
Source: Cardiovasc Ther. 2024 Dec 18;2024:1129181. doi: 10.1155/cdr/1129181 (PMC11669429; doi:10.1155/cdr/1129181)
Supplement: Supporting Information 2 — Table S2. Primers for testing the targeted genes by real-time PCR. [file 1129181.f2.pdf]

Supplementary Table 2. Primers for testing the targeted genes by real-time PCR.

Targets	Targeting Species	Forward Sequence (5'-3')	Reverse Sequence (5'-3')
TYMP	human	TGATCCGCATGAAGCGAGAC	CAGATCCATGCCCCGAAGT
GAPDH	human	AATCCCATCACCATCTTCCAG	GAGCCCCAGCCTTCTCCAT
TIMP-2 (Pair#1)	Rat	GCATCACCCAGAAGAAGAGC	GTTCAAGAAACGGACGTAGT
TIMP-2 (Pair #2)	Rat	CAAGTTCTTTGCCTGCATCA	GAACTGTAGGGAAGGACCT
MMP-2 (Pair#1)	Rat	TCCCCTGATGCTGATACTGAC	TTTTCTAACTACGGCACATGC
MMP-2 (Pair#2)	Rat	GCTGTGGACTCTAGGAGAAGGA	AAGGTGGTGCATGTTGAAACTC
TGF β1	Rat	CGCCTGCAGAGATTCAAGTC	TGACGTCAAAAGACAGCCAC
GAPDH	Rat	CAGAACATCATCCCTGCATC	CTGCTTCACCACCTTCTTGA
MMP2	Mouse	CCCGATCTACACCTACACCAA	AAACCGGTCCTTGAAGAAGAA
TYMP	Mouse	CGCGGTGATAGATGGAAGAGCA	CCGCTGATCATTGGCACCTTAC
GAPDH	Mouse	CAGCAACTCCCACTCTTCCACCTTCG	GGCCTCTCTTGCTCAGTGTCCTTGCT
