# Supplementary material for: Thymidine Phosphorylase Promotes Abdominal Aortic Aneurysm via VSMC Modulation and Matrix Remodeling in Mice and Humans
Source: Cardiovasc Ther. 2024 Dec 18;2024:1129181. doi: 10.1155/cdr/1129181 (PMC11669429; doi:10.1155/cdr/1129181)
Supplement: Supporting Information 3 — Figure S1. Flow chart of the proposed study. Figure S2. Histochemical staining images were scored based on staining intensity. We adopted a 6-point scale system, and representative images with score assignments are provided. Scale bar = 250 μm. Figure S3. TYMP expression is increased in the human AAA vessel wall. The human AAA vessel wall and healthy control aorta vessel wall (Con) were sectioned and double stained for TYMP and α-SMA. Nuclei were stained with DAPI. Scale bar = 200 μm. Figure S4. Melting curve analysis was performed to confirm the amplicons of human thymidine phosphorylase (TYMP) and glyceraldehyde 3-phosphate dehydrogenase (GAPDH). Figure S5. A. WT and Tymp−/− mice were fed a Western diet starting at 4 weeks of age for 8 weeks. Mouse body weight was monitored weekly until the implantation of the Alzet osmotic mini pump. B. Whole blood was drawn via inferior vena cava puncture at the time of sacrifice, and plasma was isolated by centrifugation using 0.109 M sodium citrate as an anticoagulant (1:9). Plasma triglyceride levels were measured using the triglyceride (TG) Colorimetric Assay Kit (EEA028, ThermoFisher Scientific). Figure S6. Necropsy of mice died after Ang II perfusion. Figure S7. Thymidine phosphorylase (TYMP) deficiency reduced the prevalence of abdominal aortic aneurysm (AAA) formation in mice. Mice were treated as mentioned in Figure 2. A Inner diameter of the abdominal aorta in the diastolic phase. B Inner diameter of the abdominal aorta in the systolic phase. Data are shown as mean ± SEM. N = 12 in WT and 11 in Tymp−/− groups for both A and B. One-way ANOVA was used to determine the time-dependent changes. C Representative echo images in the diastolic phase. D Representative echo images in the systolic phase. Figure S8. A receiver operating characteristic (ROC) analysis using the data from Figure 2(g) (abdominal aorta diameter), with the Tymp−/− group as the control and the WT group as the test group. Figure S9. Thymidine phosphorylase [file 1129181.f3.zip › Supplementary Figures.pdf]

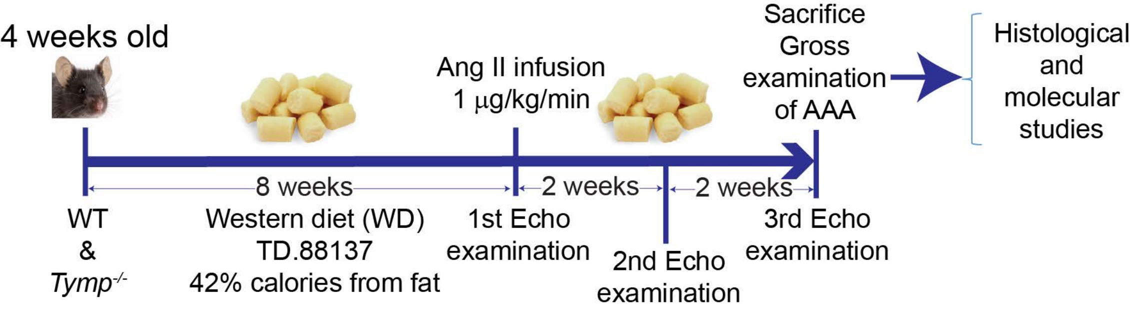


**Supplemental Figure 1. Flow chart of the proposed study.**

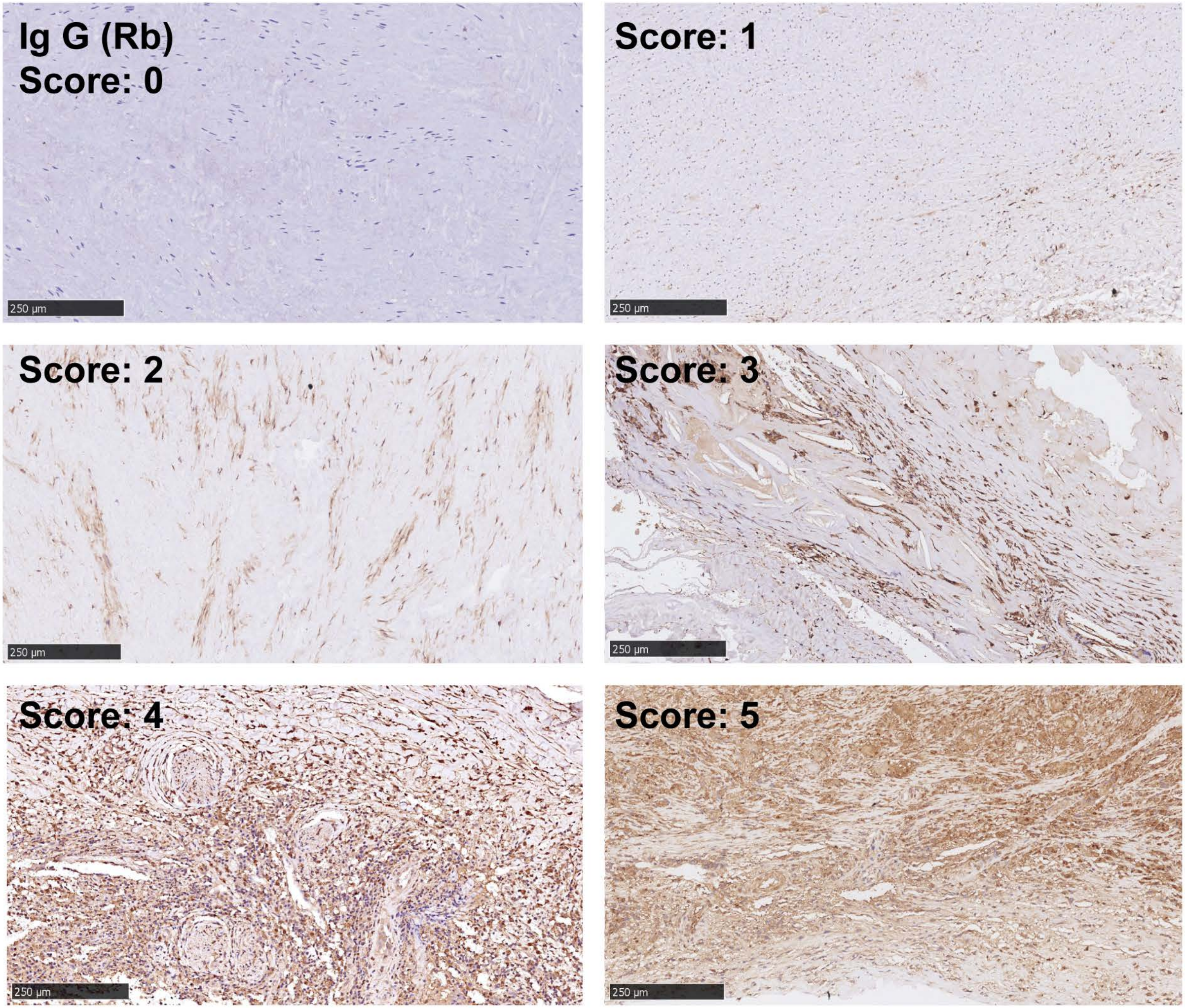


**Supplemental Figure 2:** Histochemical staining images were scored based on staining intensity. We adopted a 6-point scale system, and representative images with score assignments are provided. Scale bar = 250 $\mu$m.

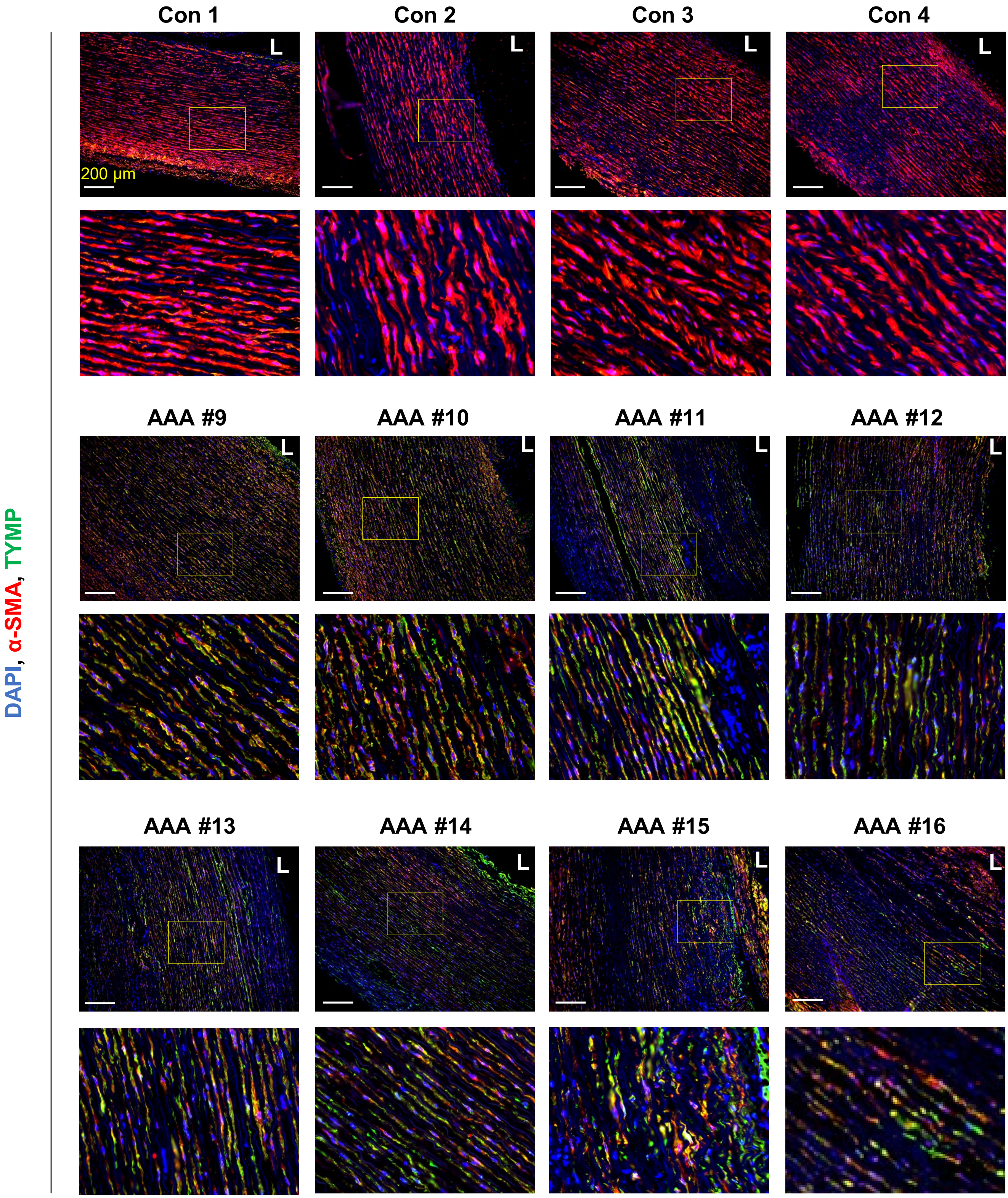


**Supplementary Figure 3. TYMP expression is increased in the human AAA vessel wall.** The human AAA vessel wall and healthy control aorta vessel wall (Con) were sectioned and double stained for TYMP and α-SMA. Nuclei were stained with DAPI. Scale bar = 200 μm.

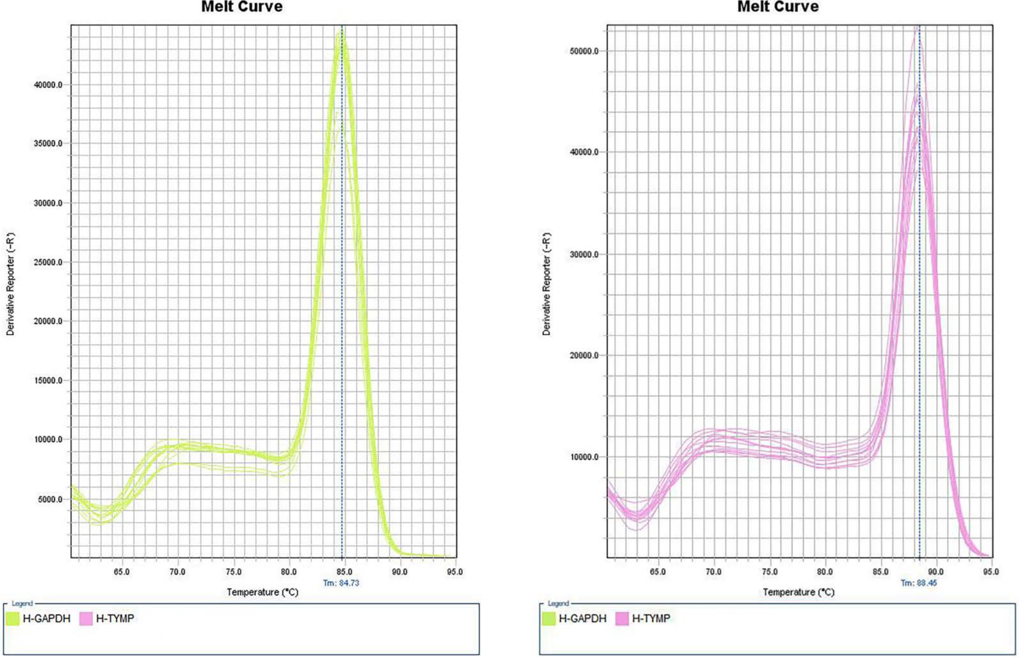


**Supplemental Figure 4.** Melting curve analysis was performed to confirm the amplicons of human thymidine phosphorylase (TYMP) and glyceraldehyde 3-phosphate dehydrogenase (GAPDH).

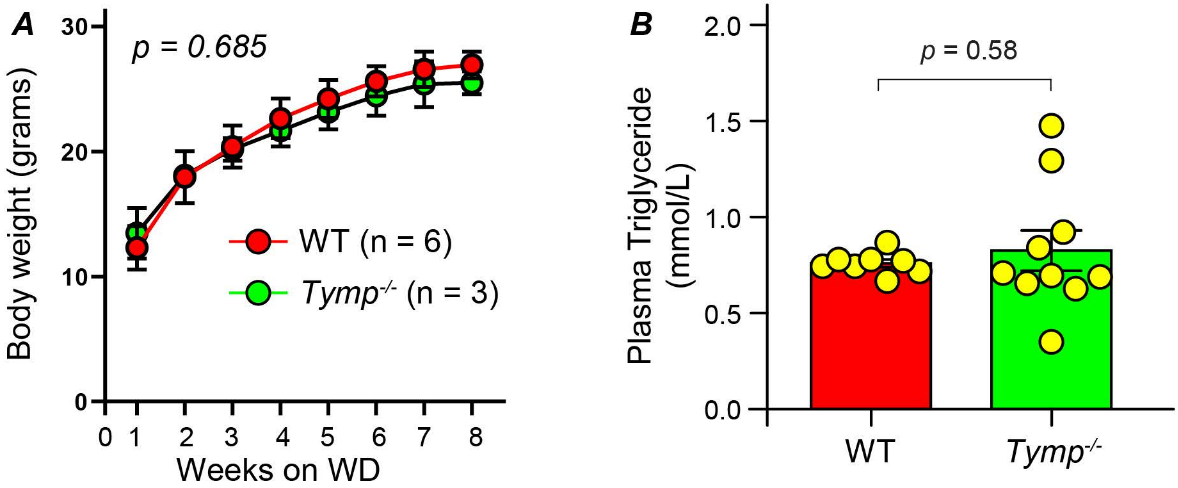


**Supplementary Figure 5. A.** WT and *Tymp*$^{-/-}$ mice were fed a Western diet starting at 4 weeks of age for 8 weeks. Mouse body weight was monitored weekly until the implantation of the Alzet® osmotic mini pump. **B.** Whole blood was drawn via inferior vena cava puncture at the time of sacrifice, and plasma was isolated by centrifugation using 0.109 M sodium citrate as an anticoagulant (1:9). Plasma triglyceride levels were measured using the Triglyceride (TG) Colorimetric Assay Kit (EEA028, ThermoFisher Scientific).

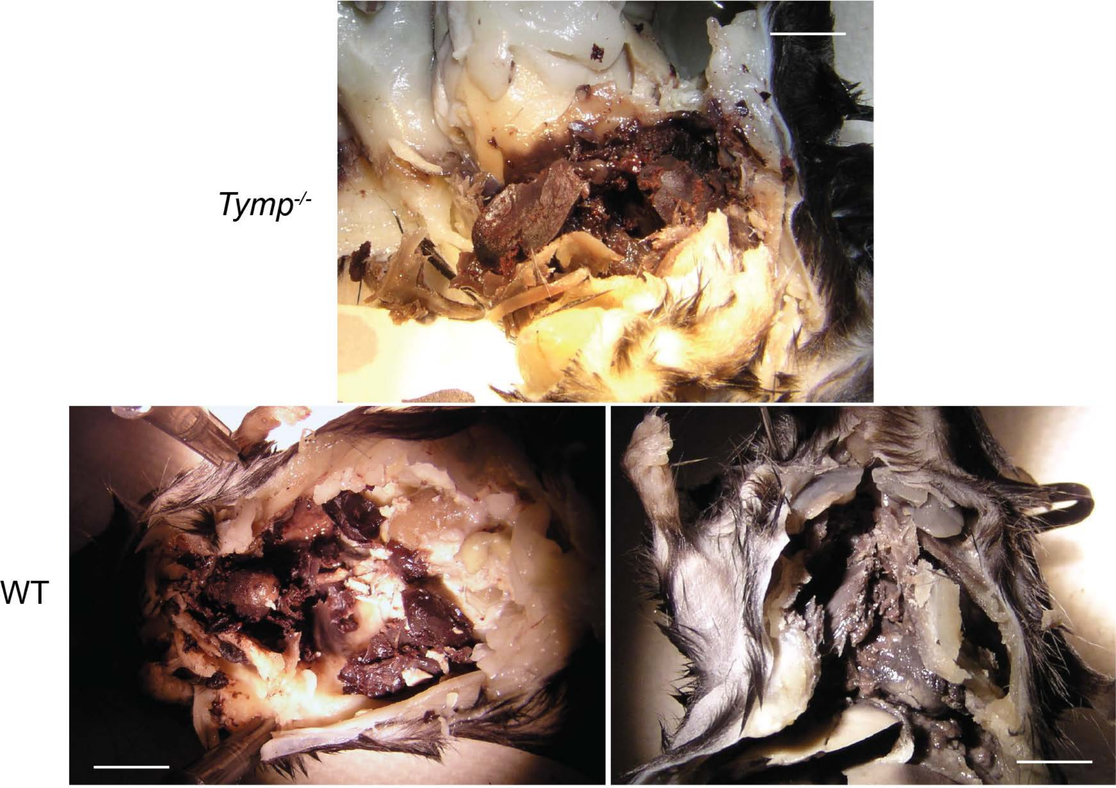


**Supplementary Figure 6.** Necropsy of mice died after Ang II perfusion.
Scale bar = 5 cm

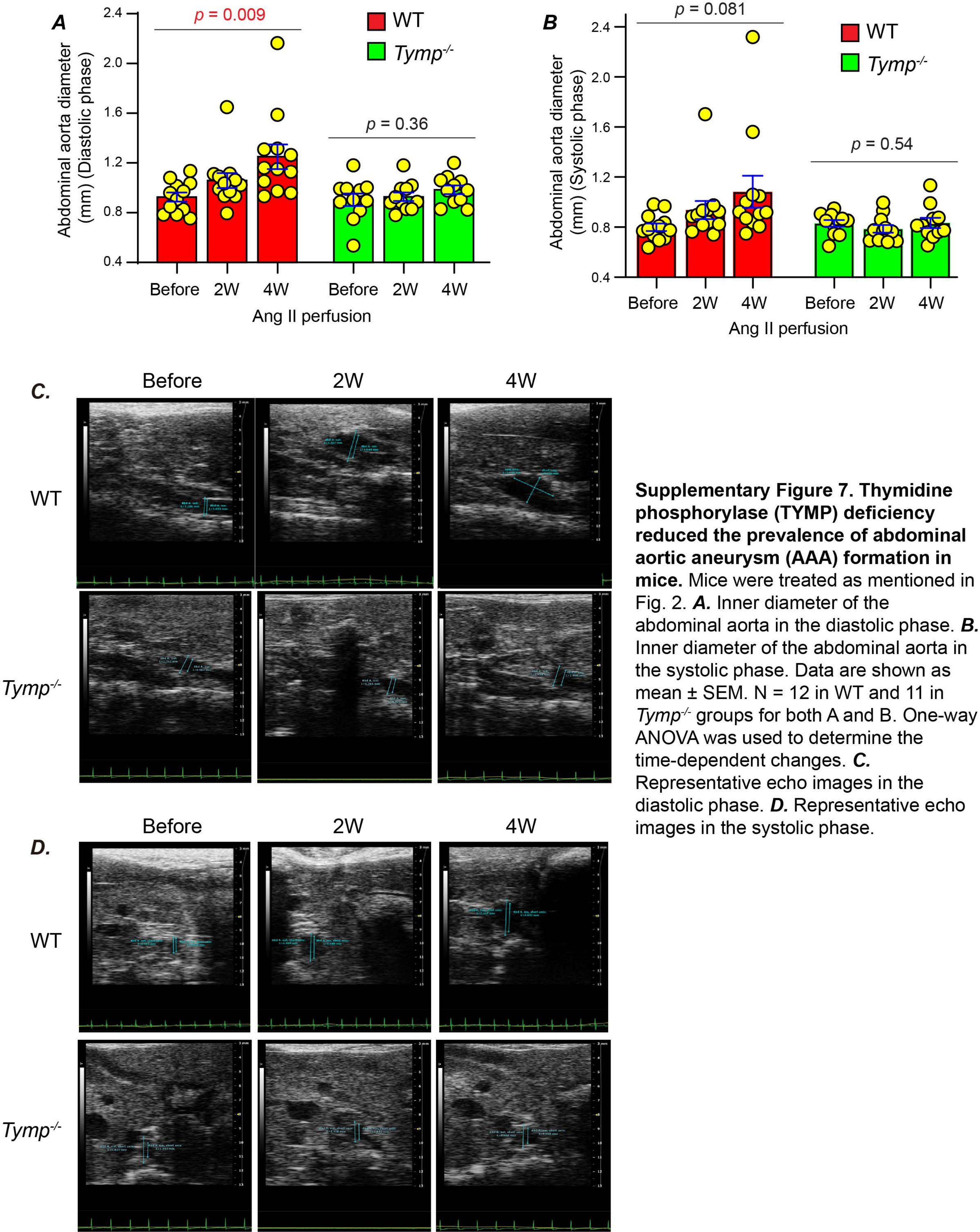


**Supplementary Figure 7. Thymidine phosphorylase (TYMP) deficiency reduced the prevalence of abdominal aortic aneurysm (AAA) formation in mice.** Mice were treated as mentioned in Fig. 2. ***A.*** Inner diameter of the abdominal aorta in the diastolic phase. ***B.*** Inner diameter of the abdominal aorta in the systolic phase. Data are shown as mean ± SEM. N = 12 in WT and 11 in *Tymp*$^{-/-}$ groups for both A and B. One-way ANOVA was used to determine the time-dependent changes. ***C.*** Representative echo images in the diastolic phase. ***D.*** Representative echo images in the systolic phase.

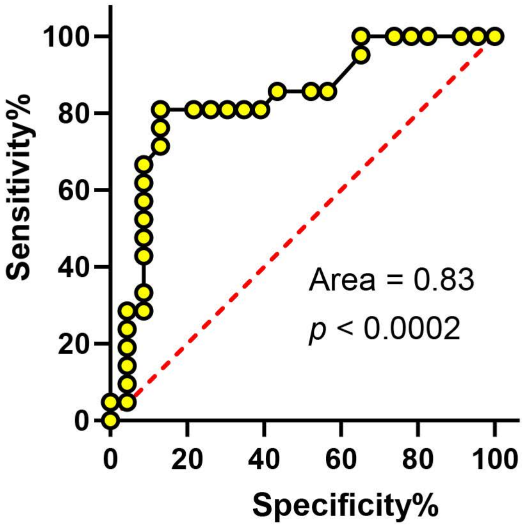


**Supplementary Figure 8.** A Receiver Operating Characteristic (ROC) analysis using the data from Figure 2G (abdominal aorta diameter), with the *Tymp*$^{-/-}$ group as the control and the WT group as the test group.

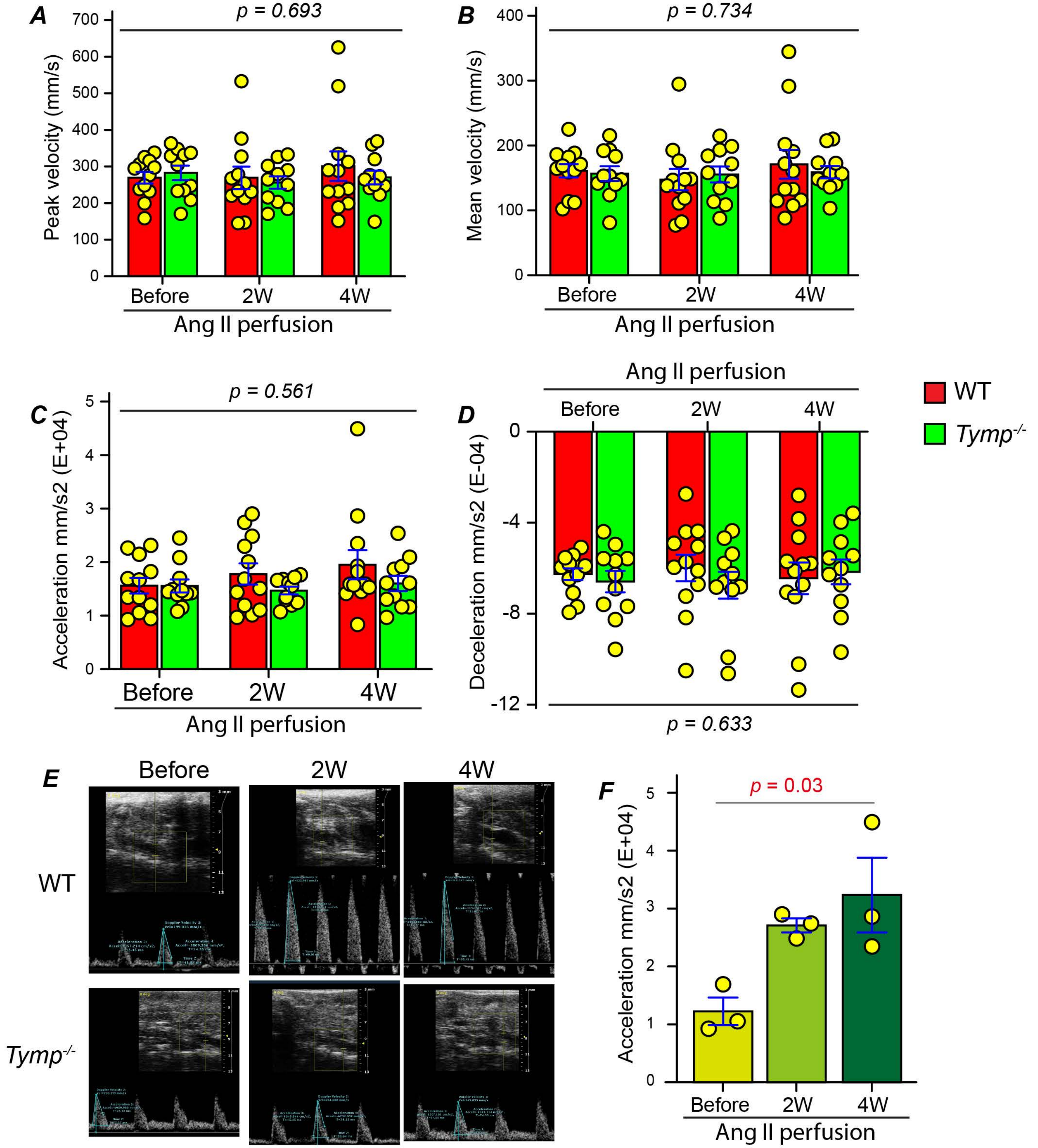


**Supplementary Figure 9. Thymidine phosphorylase (TYMP) deficiency reduced the prevalence of abdominal aortic aneurysm (AAA) formation in mice.** Mice were treated as mentioned in Fig. 2. ***A.*** Peak velocity, ***B.*** mean velocity, ***C.*** acceleration, and ***D.*** deaceleration were determined. ***E.*** Representative acceleration echo images in the WT and *Tymp*$^{-/-}$ mice. Two-way ANOVA was used to analyze the interaction between genotype and times in panels A to D. ***F.*** Statistical analysis of acceleration in the three WT mice with AAA. One way ANOVA was used. Data are shown as mean ± SEM. N = 12 in WT and 11 in *Tymp*$^{-/-}$ groups for panels A to D. Panel E represents 3 mice. N = 3 in panel F.

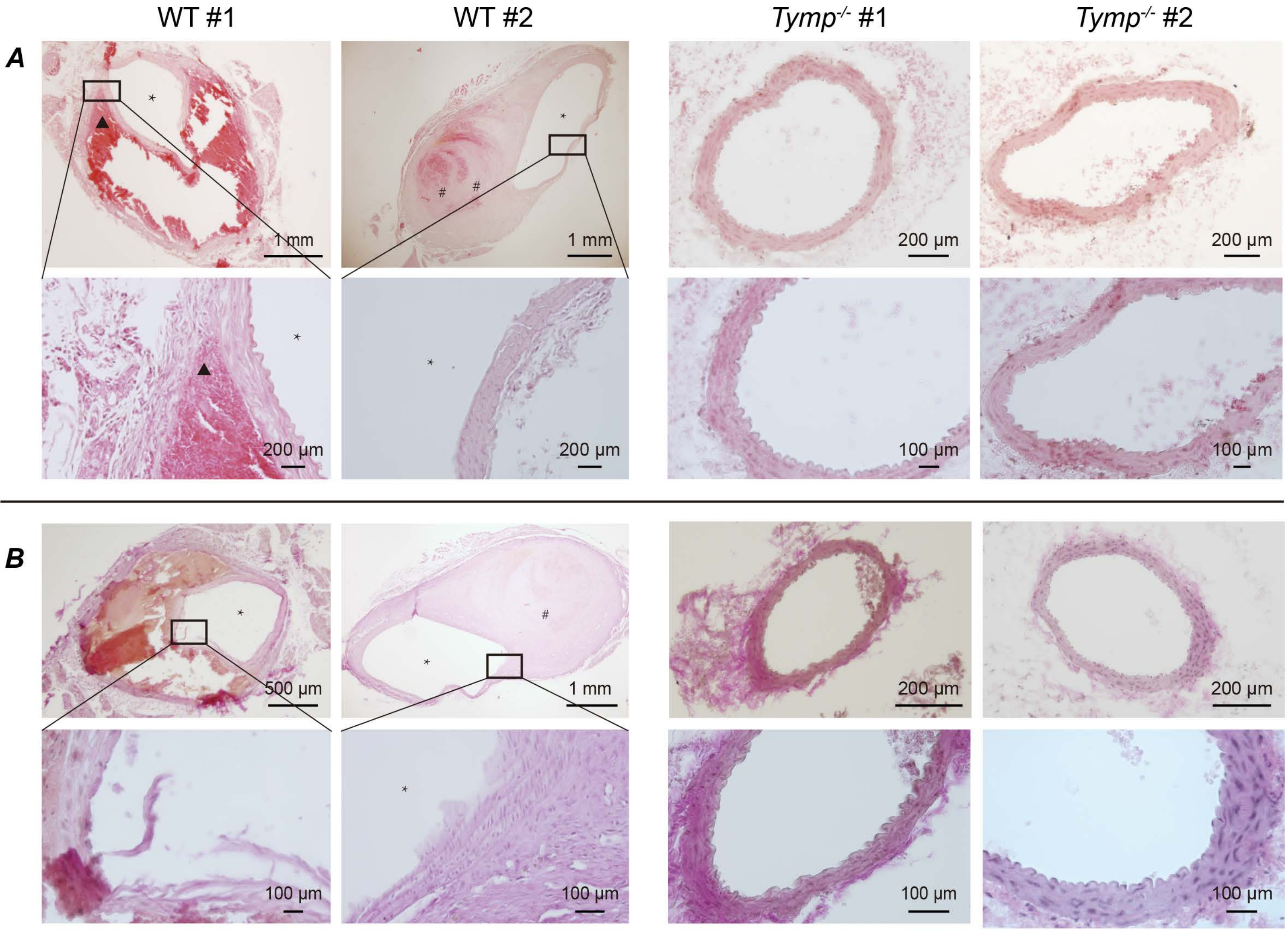


**Supplementary Figure 10. TYMP deficiency attenuates distortion of vessel wall structure in the mouse AAA model.** ***A.*** H&E staining. ***B.*** Elastica van Gieson (EVG) staining. * indicates vessel true lumen. # indicates hematoma. Images of the WT group represent 3 AAA samples, and images of the *Tymp*$^{-/-}$ group represent 6 randomly selected AAs.

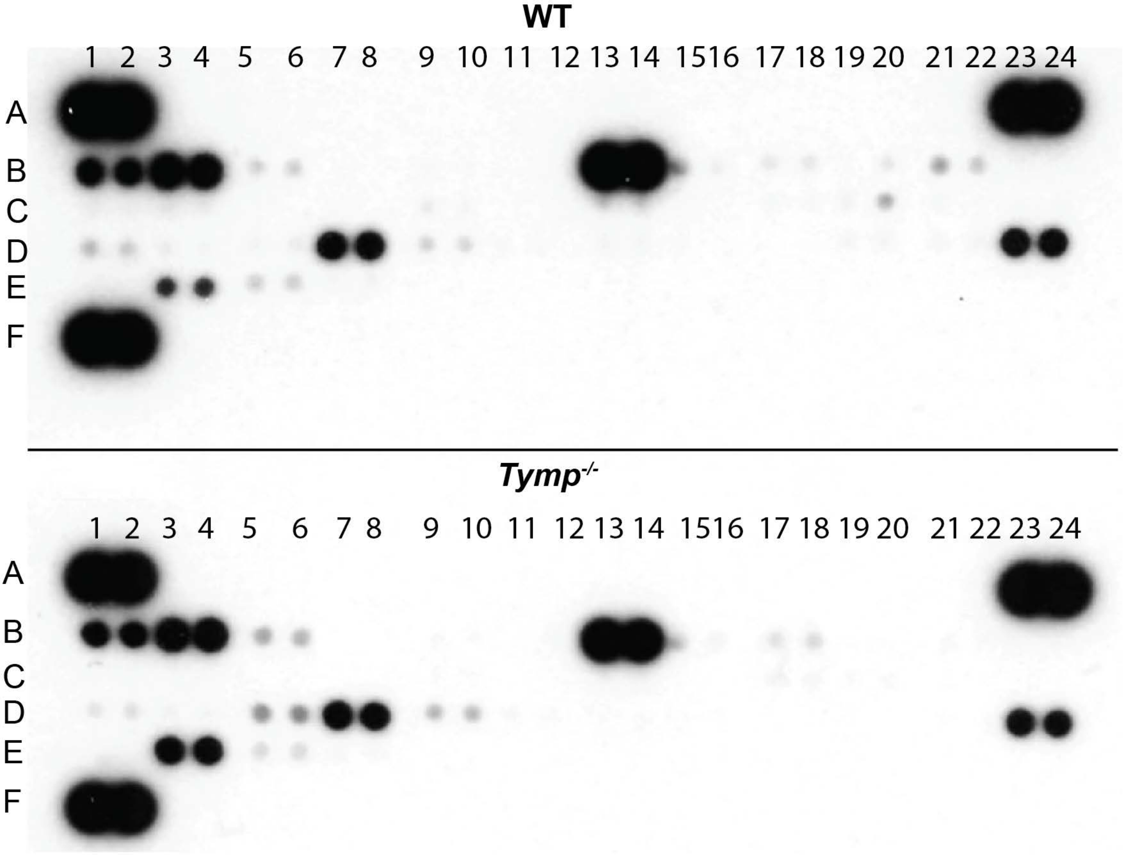


**Supplementary Figure 11. Expression of plasma cytokines in WT and *Tymp*$^{-/-}$ mice fed a Western diet and underwent Ang II treatment.** Plasma pooled from 6 WT mice (including 3 mice with AAA and 3 randomly selected) and 6 *Tymp*$^{-/-}$ mice (including 1 mouse with AAA and 5 randomly selected) were subjected to the Proteome Profiler Mouse Cytokine Array, Panel A.

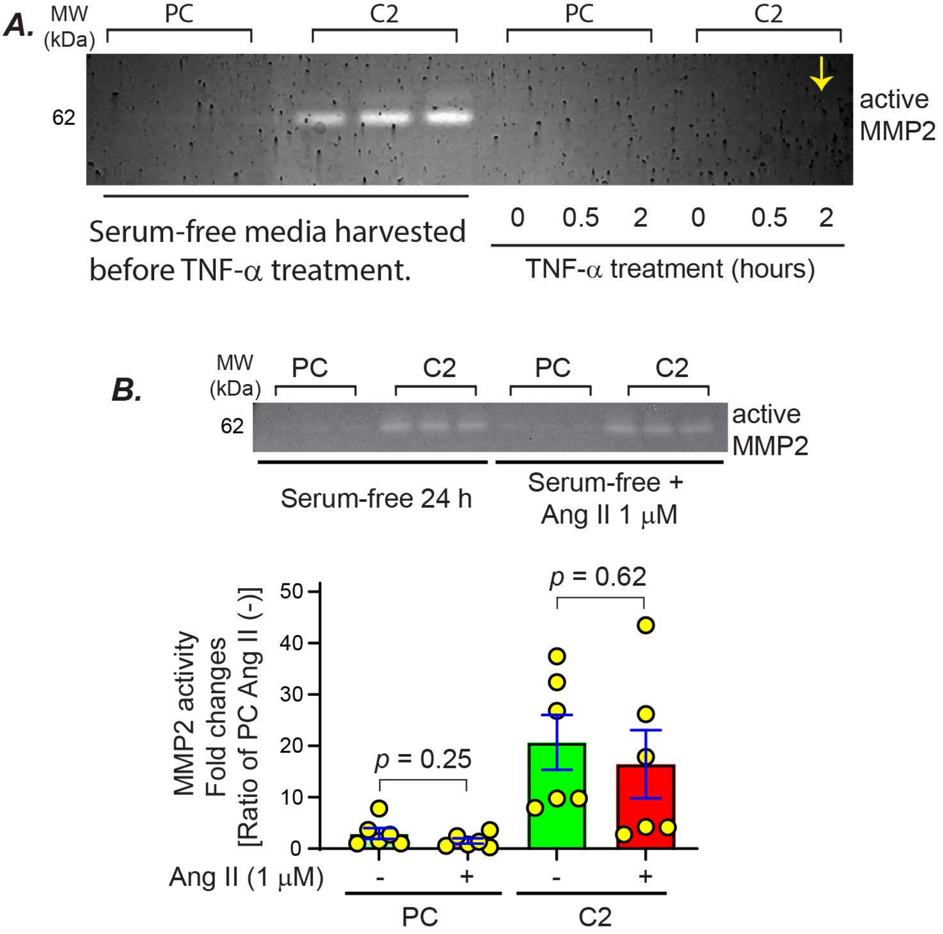


**Supplementary Figure 12. TYMP enhances MMP2 production and secretion in VSMCs.** ***A.*** C2 and PC cells (1.00E+06) were seeded into a 6-cm dish and cultured for 8 h under normal conditions. Cells were synchronized by serum starvation overnight, and then treated with TNF-α (10 ng/mL) for the indicated durations. Both the ovenight-conditioned media and media after TNF-α treatment was subjected to Zymography. The yellow arrow indicates the weak MMP2 band. Data represent two biological repeats. ***B.*** PC and C2 cells (1.00E+06) were serum-starved for 24 hours and then treated with Ang II at a final concentration of 1 µM in serum-free DMEM for additional 24 hours. The culture media were collected and used for gelatin zymography. Data are presented as fold changes of average PC before Ang II treatment. Data are shown as mean ± SEM. N = 6, The Student *t*-test was used.

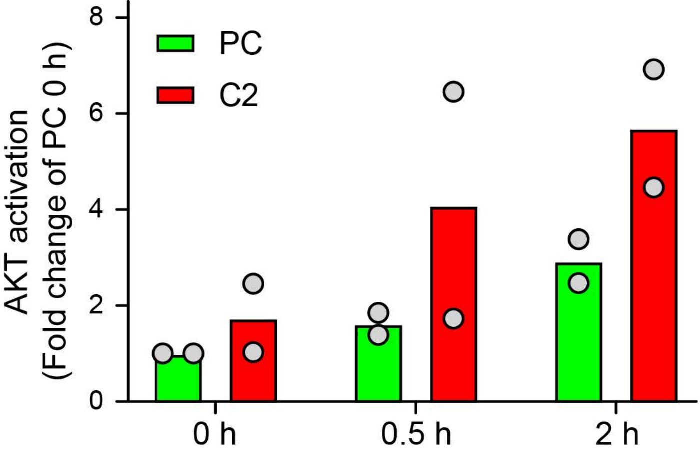


**Supplementary Figure 13. TYMP overexpression enhances AKT signaling activation in VSMCs.** Serum starvation synchronized C2 and PC cells (1.00E+06) were treated with TNF-α for the indicated durations, and cell lysates were used for western blot assay of AKT activation as shown in Figure 6C. Band intensity was analyzed using ImageJ, and p-AKT expression was adjusted with the expression of total AKT. Data were shown as fold change of PC at 0 h. Graph shows data of two biological repeats.

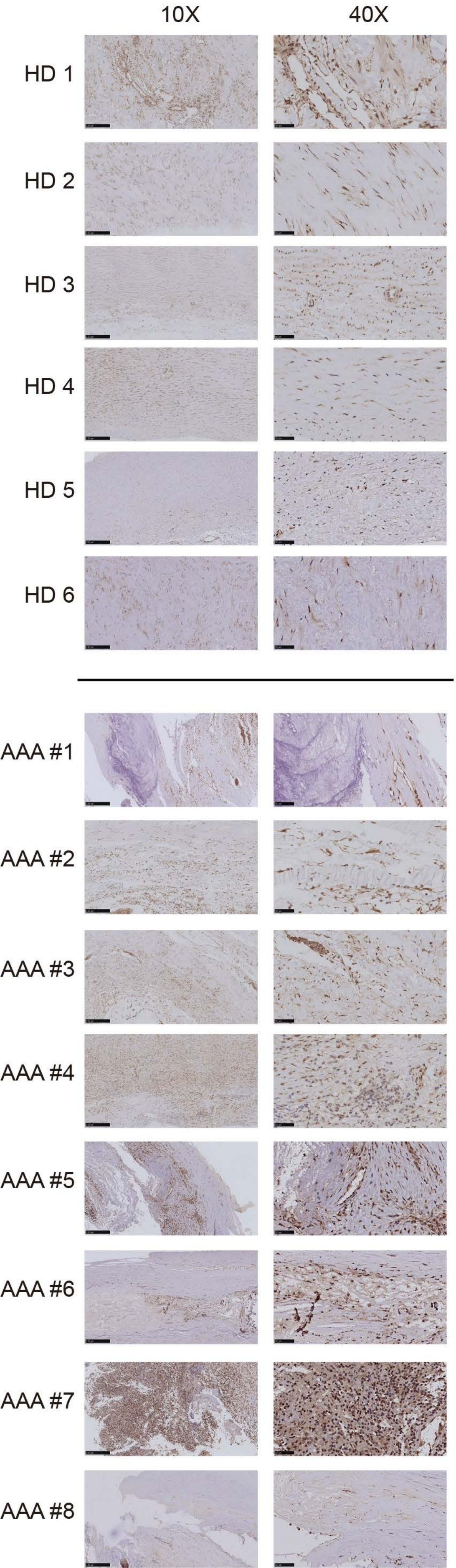


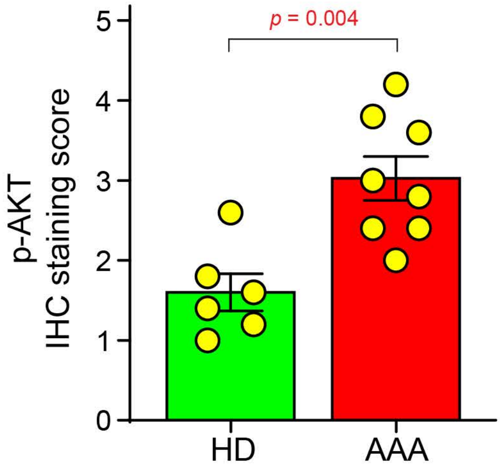


**Supplementary Figure 14. Evaluation of AKT activation in healthy human aortic vessel wall and AAA samples.** Paraffin-embedded human aortic tissues were sent to Bios Biological (https://biossci.com/), a professional company for histological study. Immunohistochemical staining of p-AKT was conducted using an antibody against phospho-pan-AKT1/2/3 (Ser473) (AF0016, Affinity), which detects endogenous levels of pan-AKT1/2/3 only when phosphorylated at Sersine 473. Positive staining is indicated by brown coloration, while nuclei were stained with hematoxylin (blue). The images were scanned and scored by lay individuals based on staining intensity showed in Supplementary Figure 1, and score data were used for statistical analysis, with results presented in bar graph format. Data are shown as mean ± SEM. N = 6 in the Health Donor (HD) group and 8 in the AAA group. The Student *t*-test was used. Scale bar = 250 μm in 10X images, and 50 μm in 40X images.

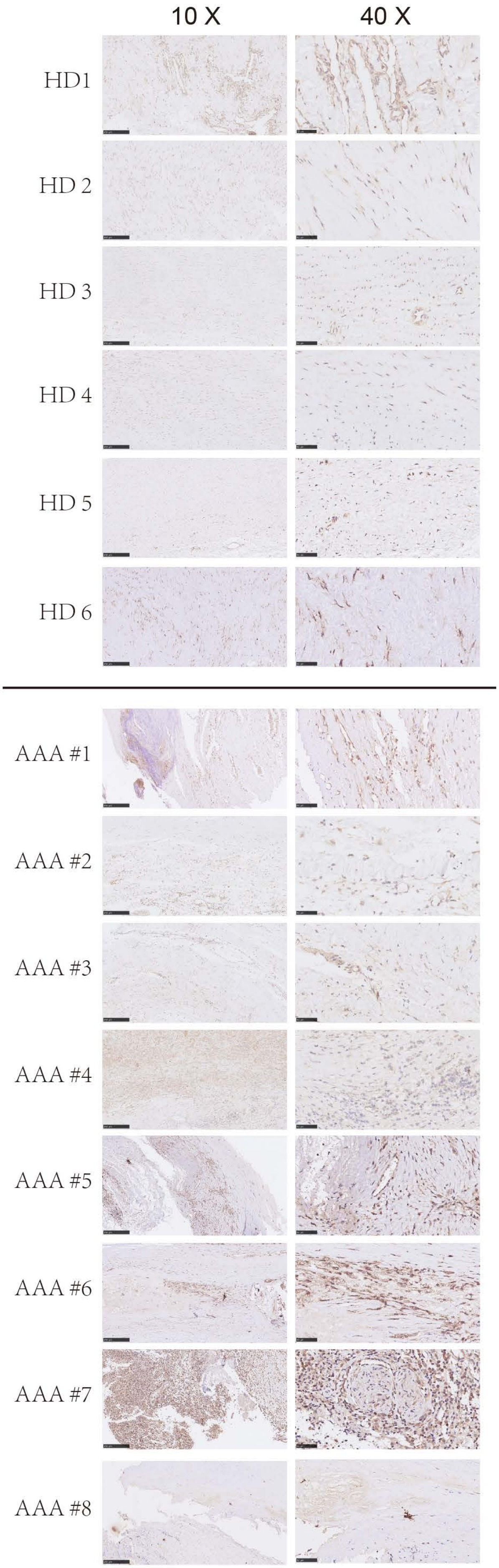


**Supplementary Figure 15.** IHC of TGFβ1 in human healthy donor (HD) aorta or in the AAA vessel walls. Brown indicates positive staining. Scale bar = 250 μm in 10X images, and 50 μm in 40X images.

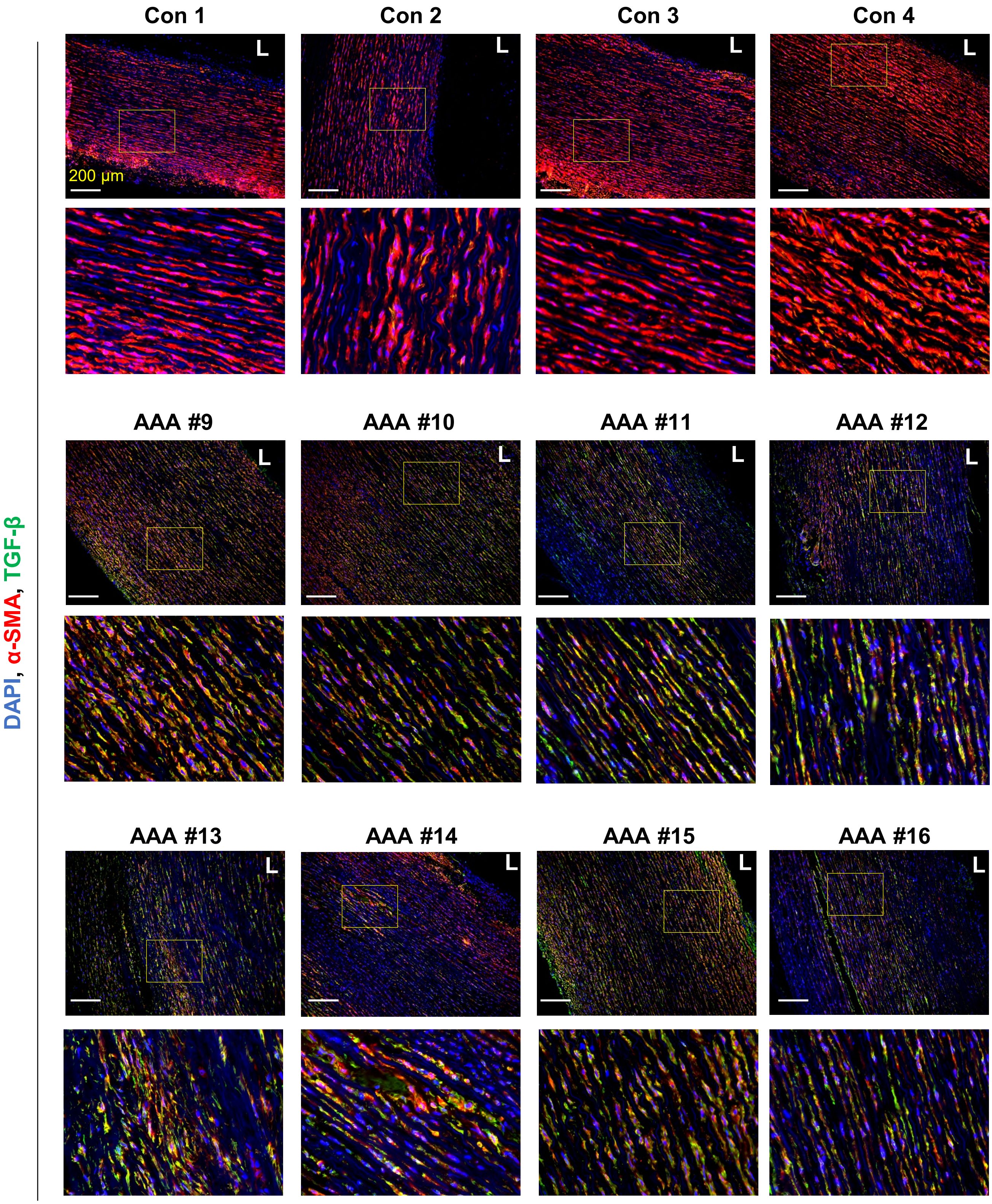


**Supplementary Figure 16. TGFβ1 expression is increased in the human AAA vessel wall.** The human AAA vessel wall and healthy control aorta vessel wall (Con) were sectioned and double stained for TGFβ1 and α-SMA. Nuclei were stained with DAPI. Scale bar = 200 μm.

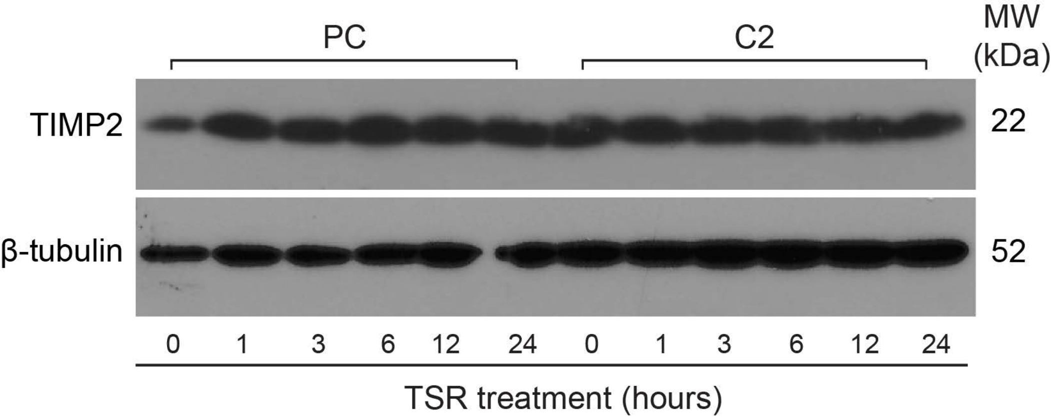


**Supplementary Figure 17. TYMP overexpression does not affect TIMP2 expression in VSMCs.** As mentioned in Fig. 7G, serum-starved PC and C2 cells (1.00E+06) were treated with serum-free media in the presence of 10 μg/mL TSR for the indicated durations. TIMP2 levels were examined by western blot, with β-tubulin blotted as loading control.
