# Supplementary material for: Thymidine Phosphorylase Promotes Abdominal Aortic Aneurysm via VSMC Modulation and Matrix Remodeling in Mice and Humans
Source: Cardiovasc Ther. 2024 Dec 18;2024:1129181. doi: 10.1155/cdr/1129181 (PMC11669429; doi:10.1155/cdr/1129181)

Fig.4 A, MMP2 Zymography

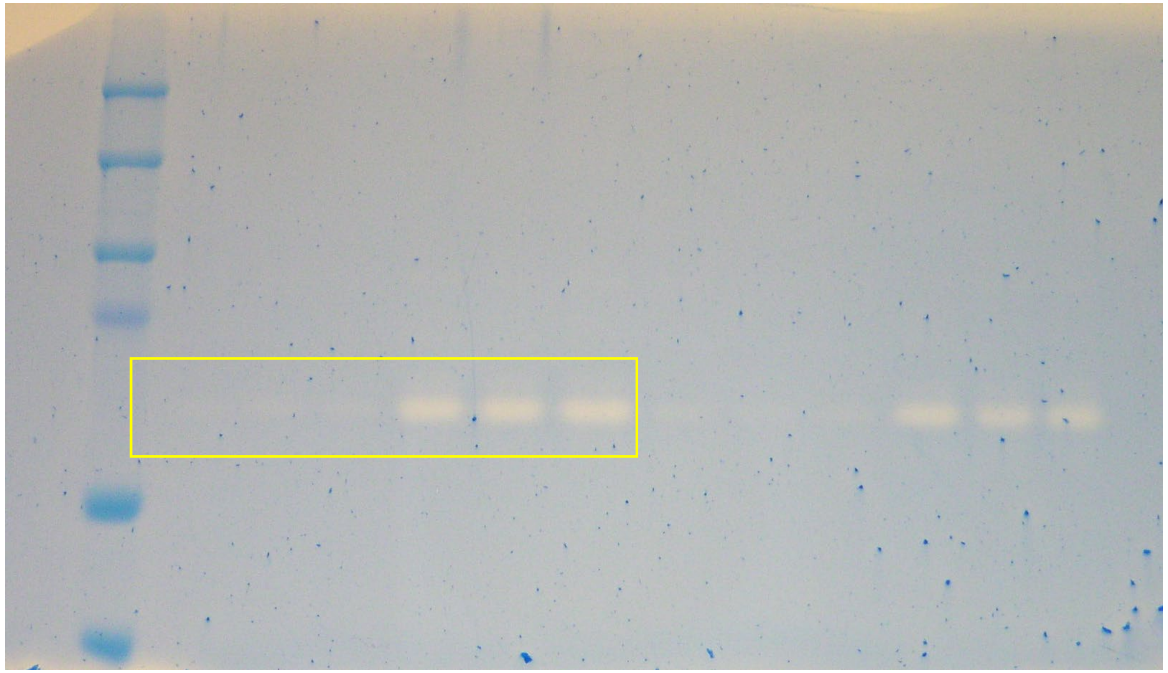

Fig. 4B, MMP-2

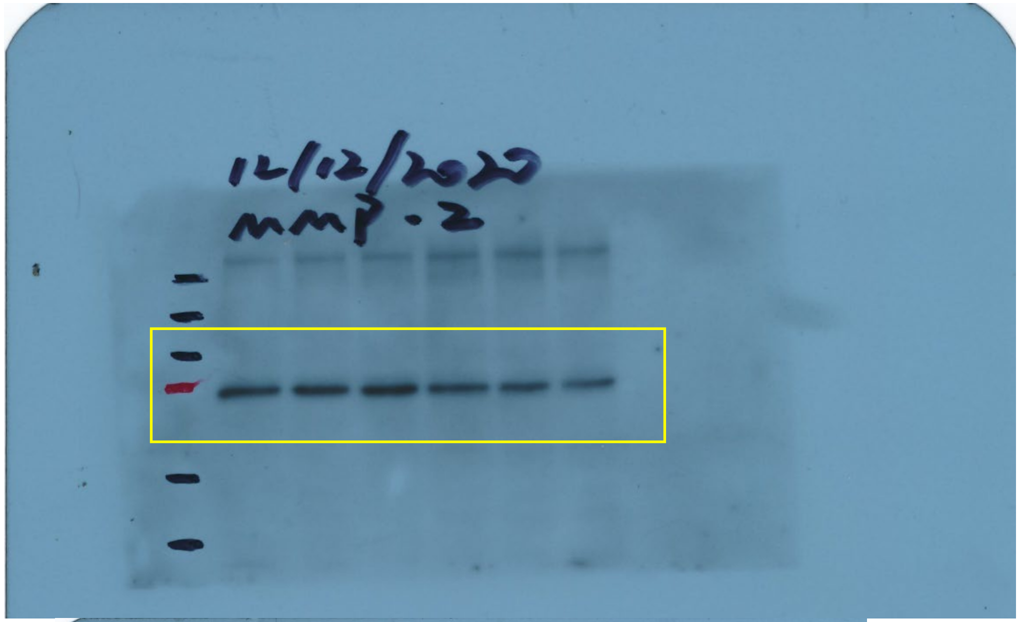

Fig. 4B, pan-actin

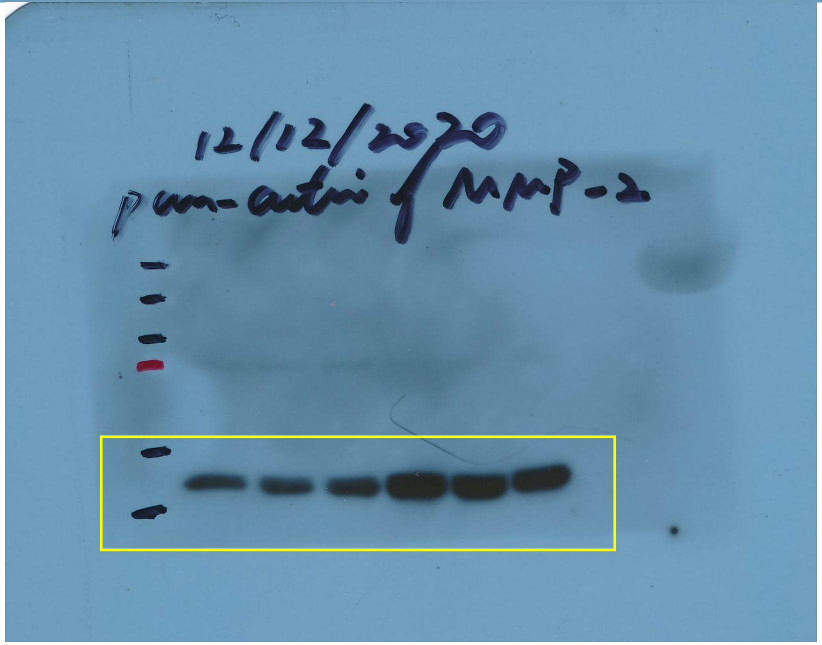

Fig. 4C, MMP2

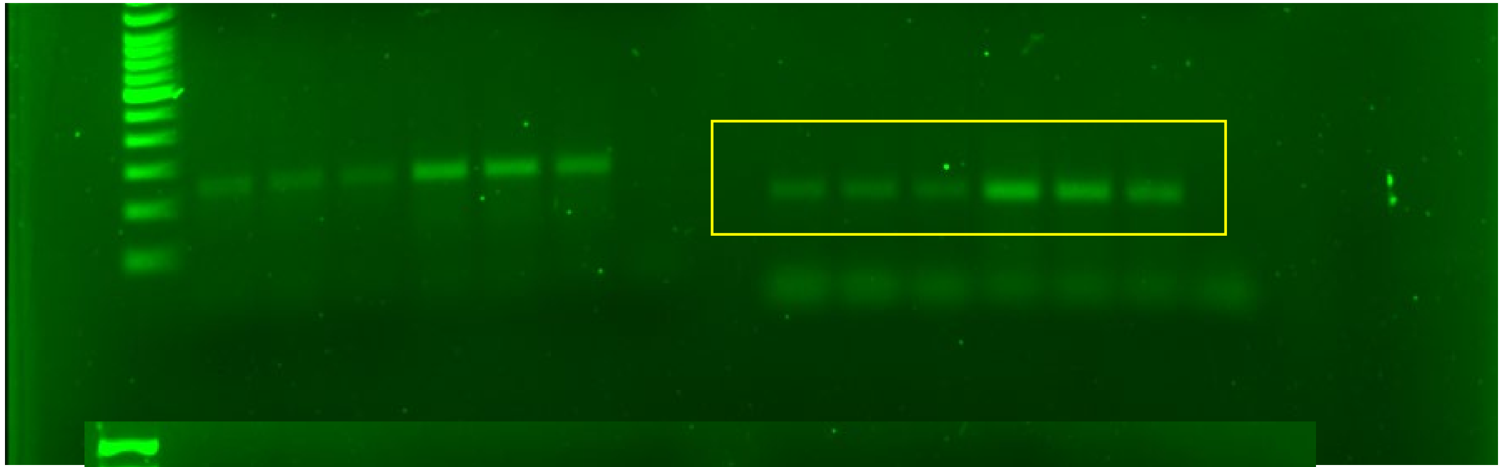

Fig. 4C, TIMP2

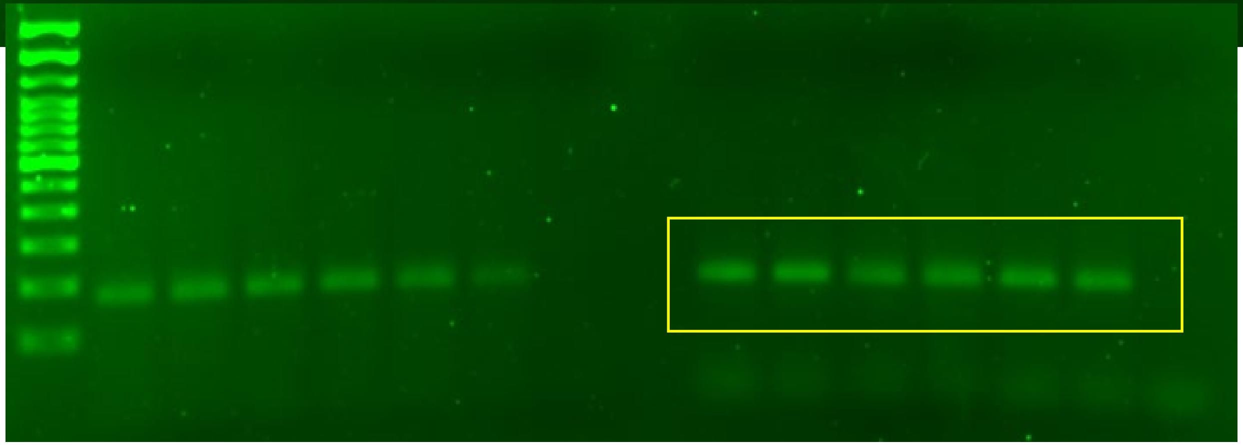

Fig. 4C, GAPDH

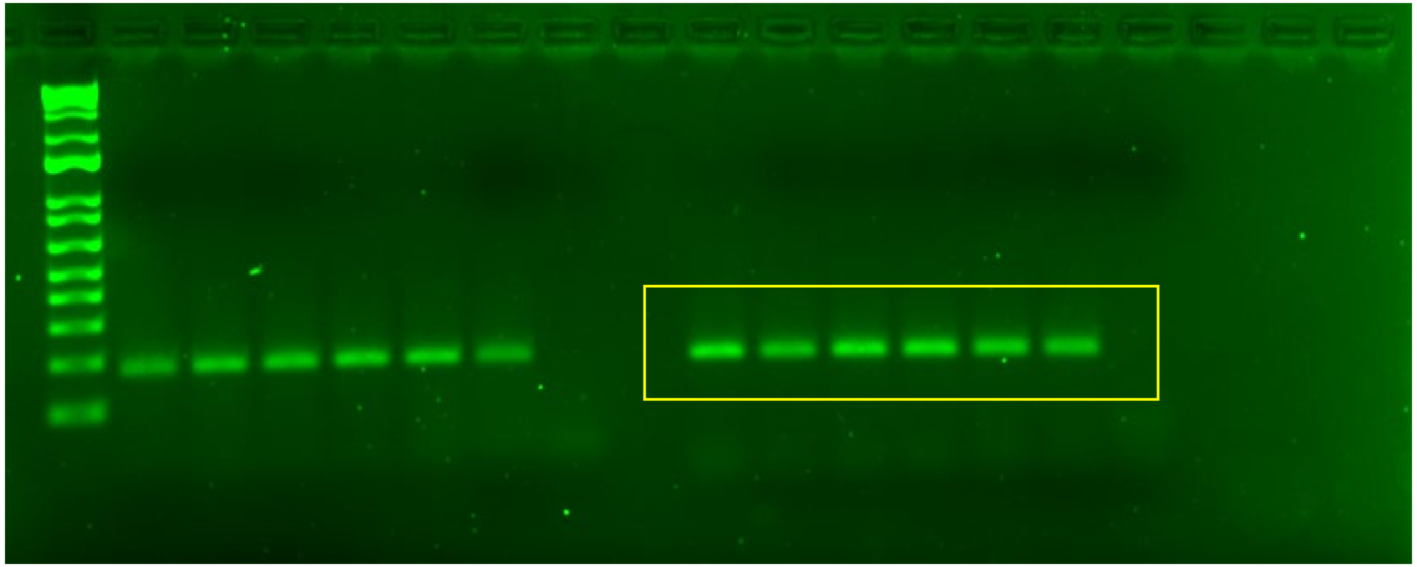

Fig. 4E, MMP2 activity

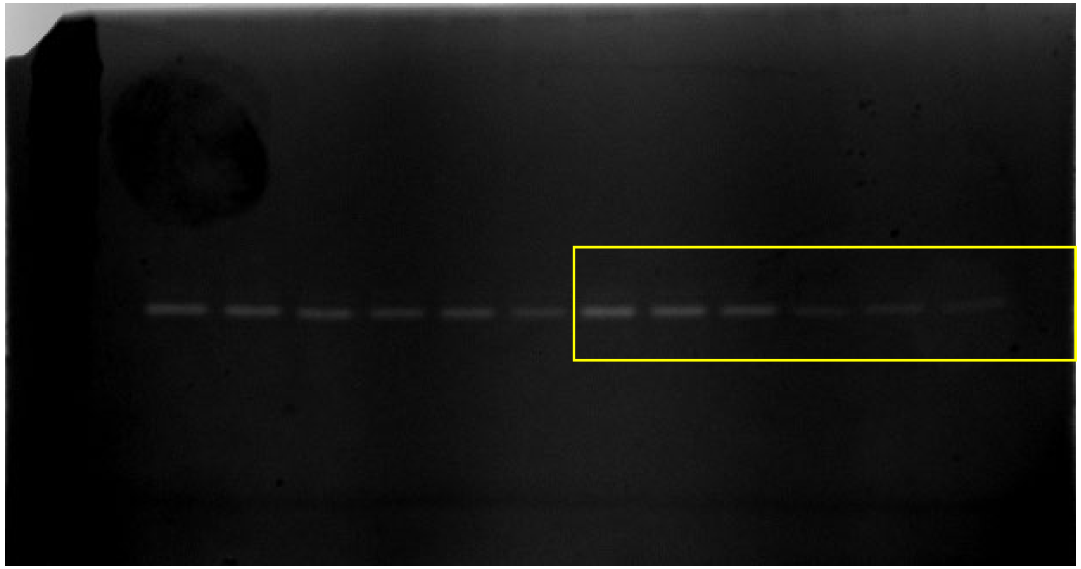

Fig. 4G, MMP2, Western blot

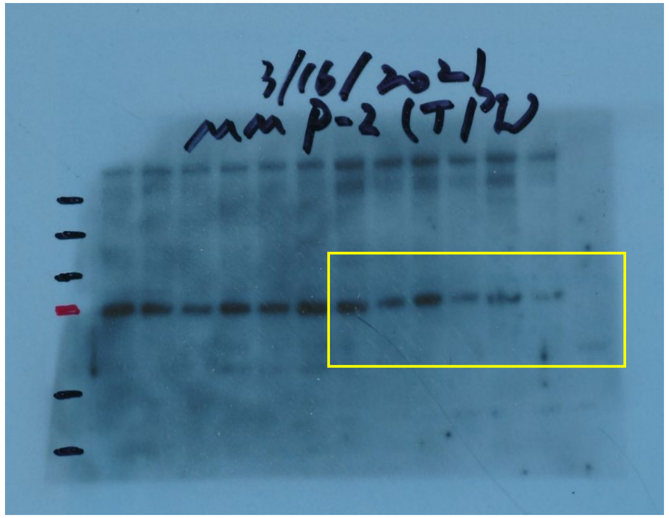

Fig. 4G, TYMP, Western blot

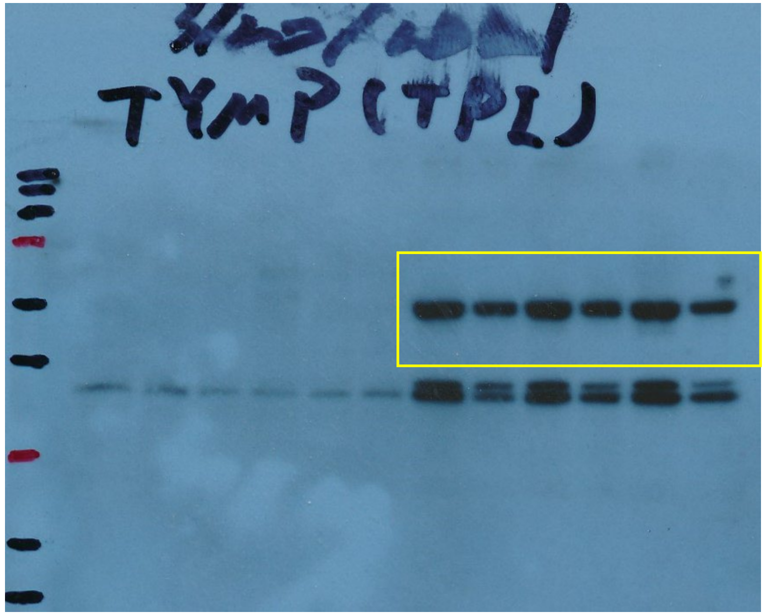

Fig. 4G, α-tubulin Western blot

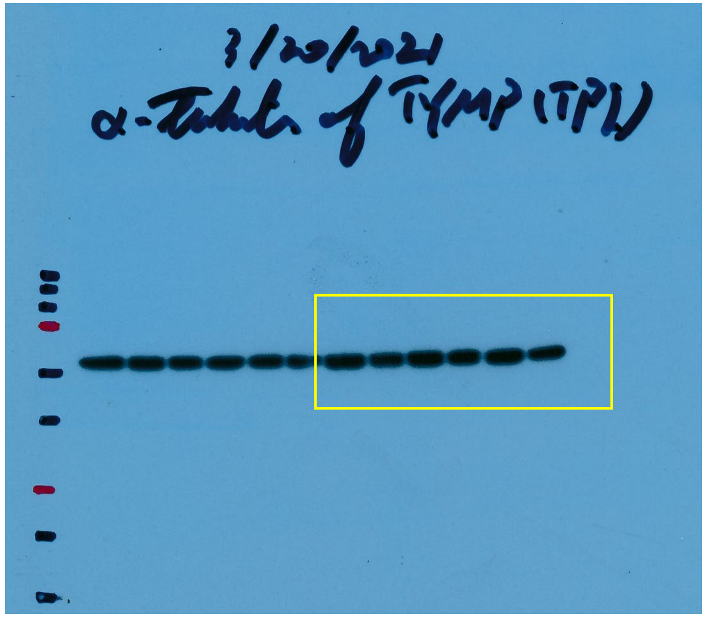

Fig. 4H, MMP2 zymography

Zymography working sheet

Date:	2 / 6 / 2021	Investigator:	Liang Hong					Gel:	# 2
Aim	MMP activity of TPL_PC & C2 (20210921) - TPL medium 24h (6.25, 12.5, 25, 50, 100 μm)								
Gel Concentration:	7.5% SDS-PAGE + 1‰ Gelatin								

Lane 1	2	3	4	5	6	7	8	9	10	11	12	13	14	15
Sample buffer	Marker	PC ① Ctrl	PC ② 6.25 μM	PC ③ 12.5 μM	PC ④ 25 μM	PC ⑤ 50 μM	PC ⑥ 100 μM	C2 ① Ctrl	C2 ② 6.25 μM	C2 ③ 12.5 μM	C2 ④ 25 μM	C2 ⑤ 50 μM	C2 ⑥ 100 μM	Sample buffer

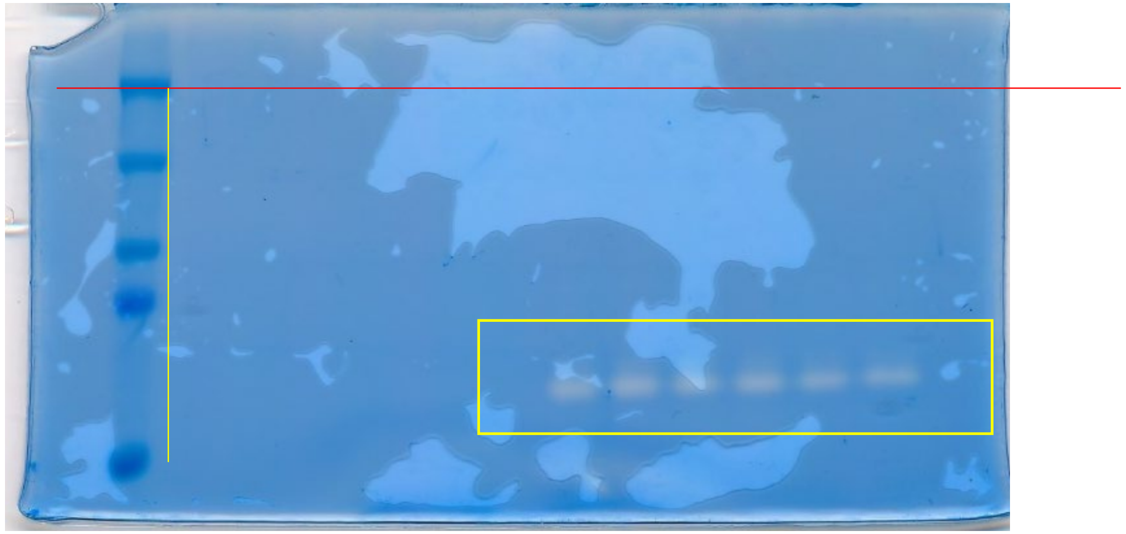

Fig.5 B: MMP-2 and Pan-actin

Membran #1

Dr. Wei Li Lab---Western Blot working sheet

pro: 33.95 μg/lane

Data:	9/16/2020	Investigator	Liang Hong
Aim	Expression of MMP-2, CTGF, TYMP in PC & C2 treated with TNF-α media (9/13)		
Gel Concentration:	7.5% SDS-PAGE		

strip 9/17

1st antibody:	MMP-2. Santa. Cruz. Mouse	Concentration:	1:500	Buffer:	5% skimmed milk
1st antibody:	CTGF. Santa. Cruz. Mouse	Concentration:	1:500	Buffer:	

Lane 1	Lane 2	Lane 3	Lane 4	Lane 5	Lane 6	Lane 7	Lane 8	Lane 9	Lane 10	Lane 11	Lane 12	Lane 13	Lane 14	Lane 15
Marker	PC Ctrl	C2 Ctrl	PC Ctrl	PC 0.5h	PC 2h	C2 Ctrl	C2 0.5h	C2 2h	Sample Buffer					

Loading control: pan-actin 1:10000

1st Ab: TYMP. Abcame. Rabbit. 1:1000

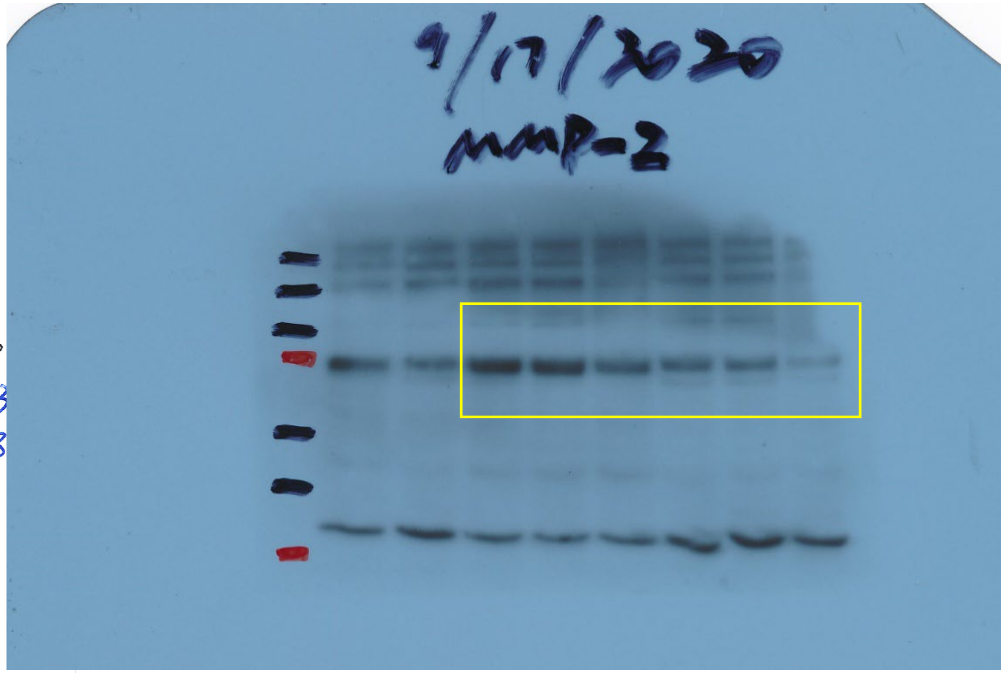

pro
63
38

45KD
50KD

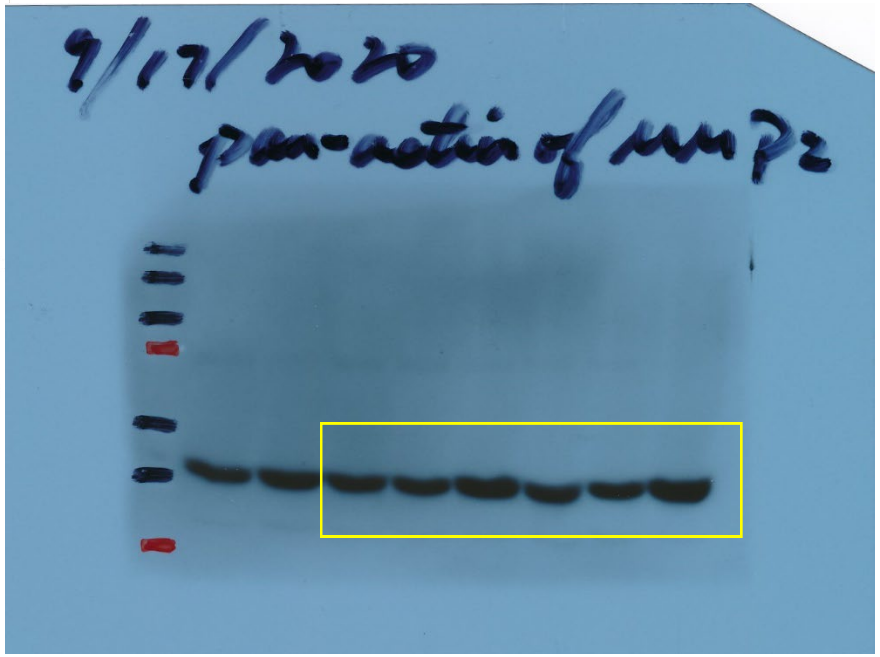

Fig. 5C

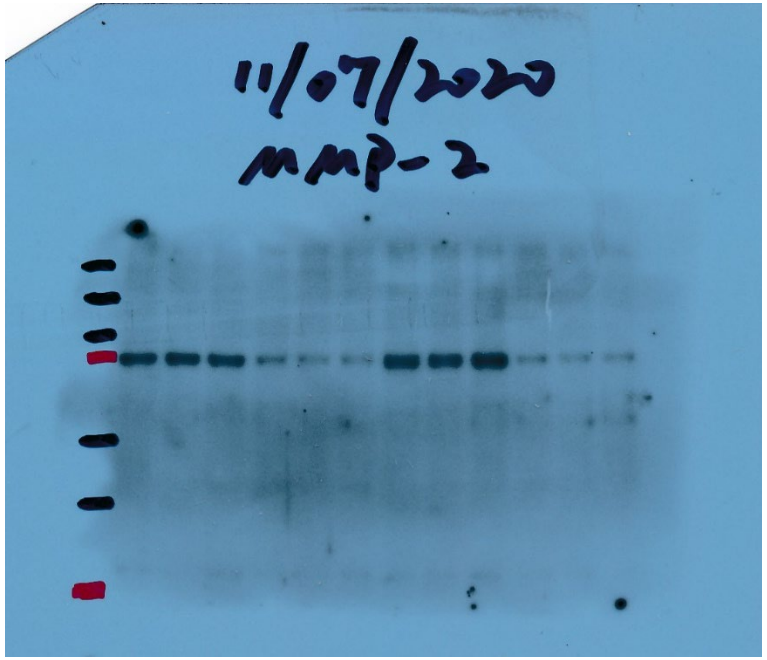

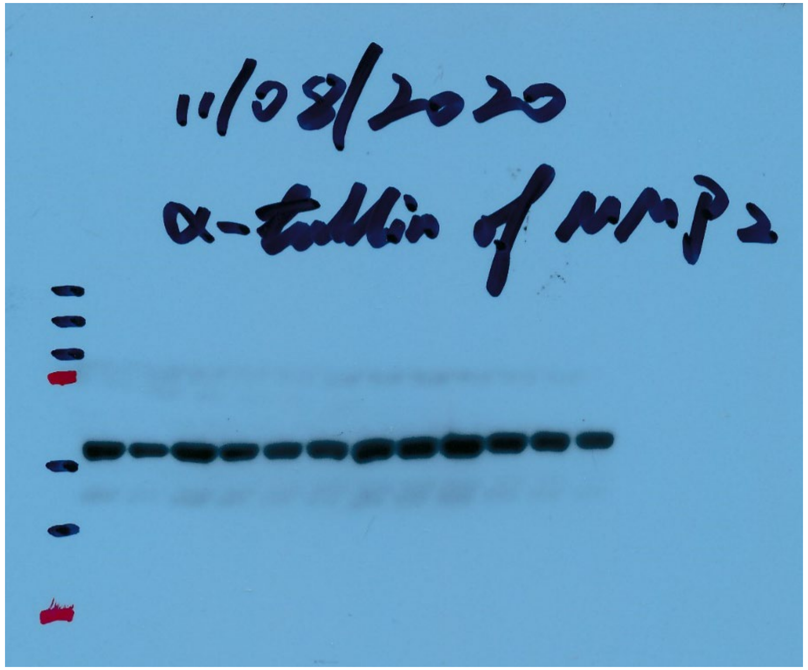

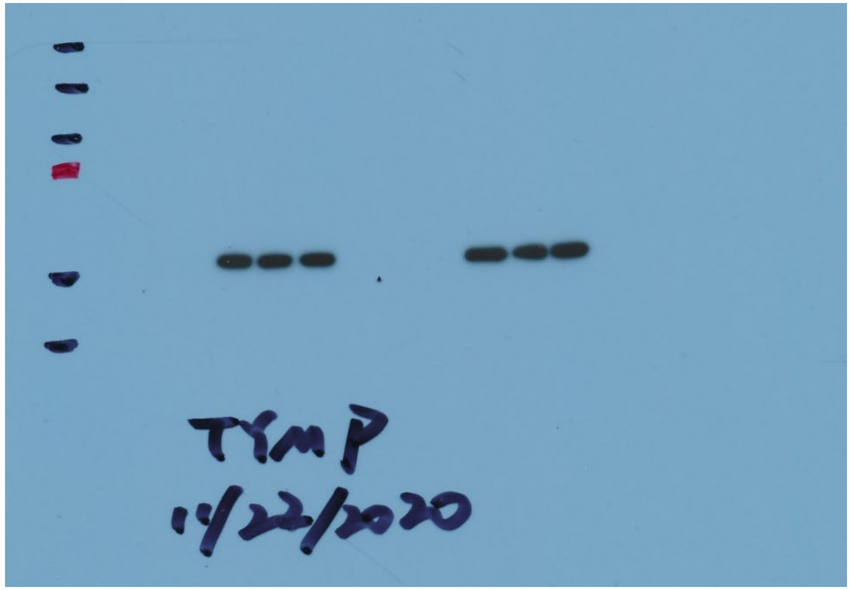

Fig. 6B

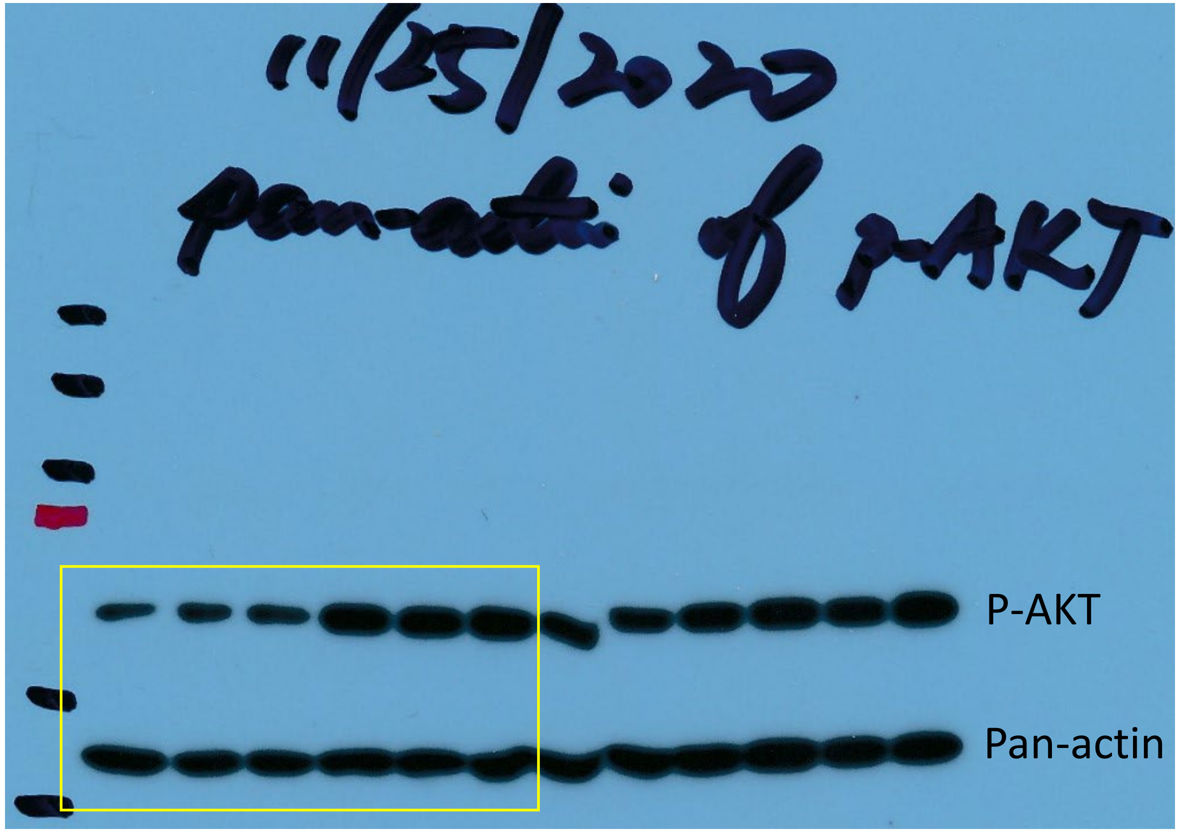

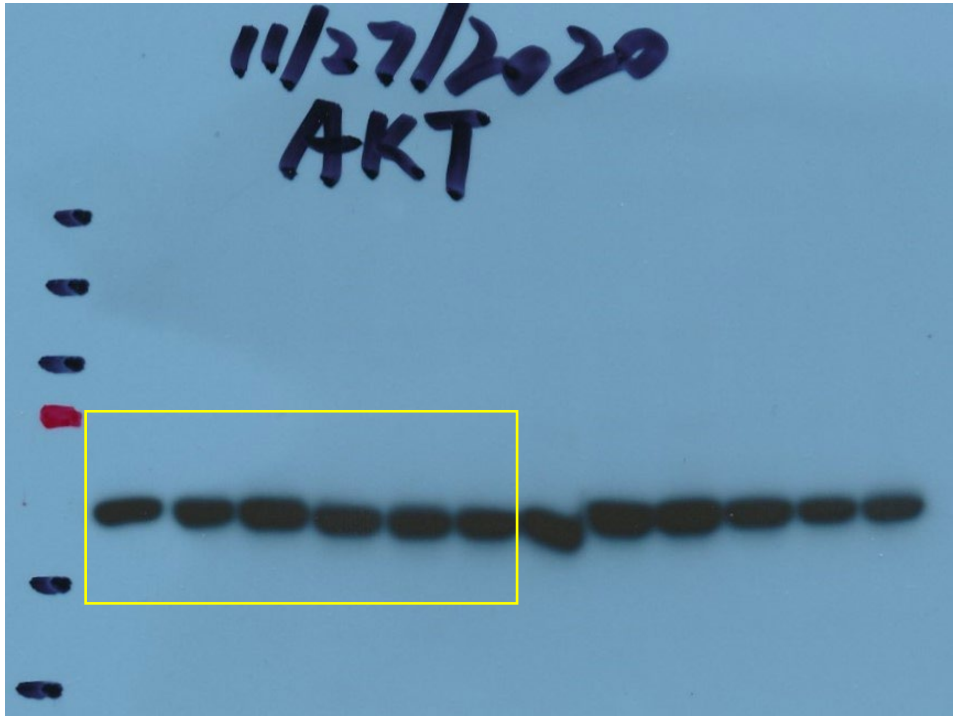

Fig.6 C

Membrane #2

Dr. Wei Li Lab---Western Blot working sheet

pro: 33.95 μg/lane

Data:	9/16/2020	Investigator	Liang Hong
Aim	Expression of p-AKT, AKT in PC & C2 treated with TNF-α media (9/13)		
Gel Concentration:	7.5% SDS-PAGE		

strip 9/17

1st antibody:	p-AKT, Cell Signaling Rabbit	Concentration:	1:2000	Buffer:	5% skimmed milk
1st antibody:	AKT, Cell Signaling Mouse	Concentration:	1:2000	Buffer:	

60KD

Lane 1	Lane 2	Lane 3	Lane 4	Lane 5	Lane 6	Lane 7	Lane 8	Lane 9	Lane 10	Lane 11	Lane 12	Lane 13	Lane 14	Lane 15
Marker	PC Ctrl	C2 Ctrl	PC Ctrl	PC 0.5h	PC 2h	C2 Ctrl	C2 0.5h	C2 2h	Sample buffer					

Loading Control: pan-actin 1:10000

45KD

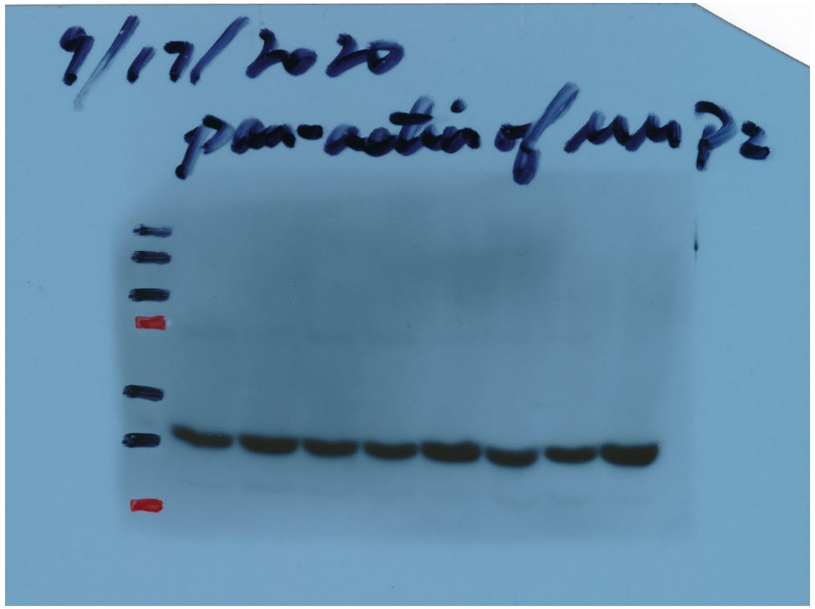

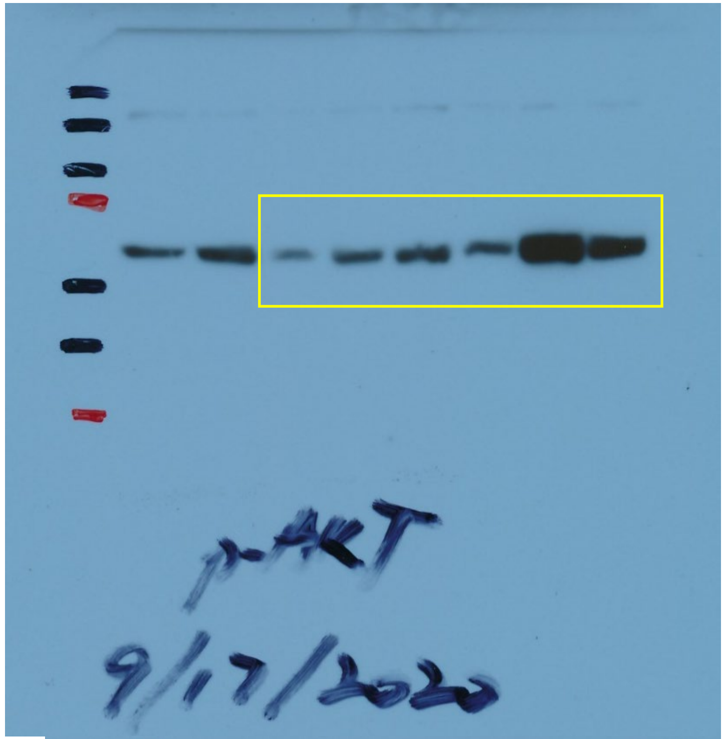

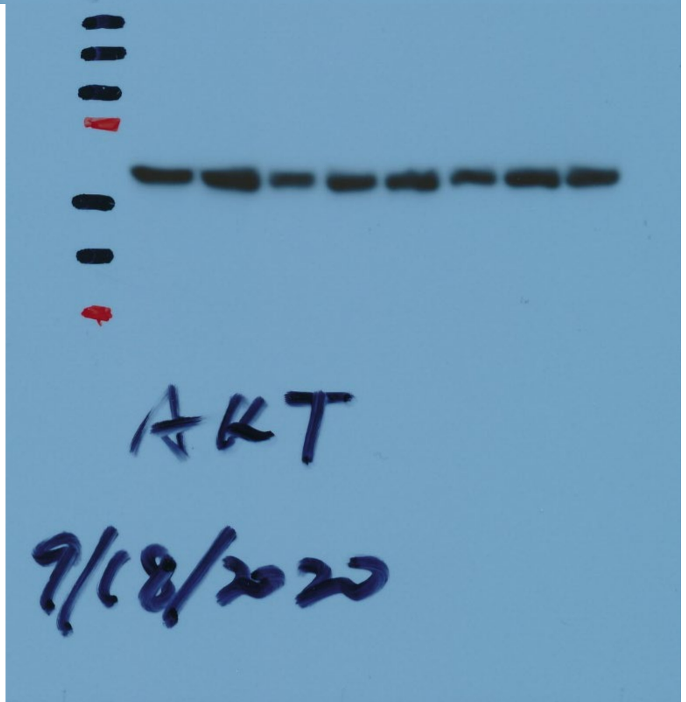

Fig. 7D

Dr. Wei Li Lab ---- Western Blot working sheet

Date:	11 / 01 / 2020	Investigator:	Liang Hong
Membrane:	# 2		
Aim	p-JNK, CTGF, TGFβ1 in PC & C2 treated Ang II (1μM, 24h, 10/30/2020)		
Gel Concentration:	7.5% SDS-PAGE	Protein weight:	17.44 μg / Lane

1st Antibody:	p-JNK, Cell Signaling Rabbit	Concentration:	1 : 1000	Buffer: 5% skimmed milk	JNK2 54, JNK1 46 KD
2nd AB	anti- Rabbit HRP	Concentration:	1 : 2000		
11/05 1st Antibody:	~~p-Src (Tyr416)~~ TGF Santa Cruz Ms	Concentration:	1 : 500 ~~1000~~	Buffer: 5% skimmed milk	25 ~~60~~ KD
2nd AB	anti- Mouse HRP	Concentration:	1 : 2000		
Loading Control:	pan-actin	Concentration:	1 : 10000		45 KD

Lane 1	2	3	4	5	6	7	8	9	10	11	12	13	14	15
Sample Buffer	Marker	PC Ctrl ①	PC Ctrl ②	PC Ctrl ③	PC AngII ④	PC AngII ⑤	PC AngII ⑥	C2 Ctrl ①	C2 Ctrl ②	C2 Ctrl ③	C2 AngII ④	C2 AngII ⑤	C2 AngII ⑥	Sample Buffer

11/06 TGFβ1 Santa Cruz 1:500, Mouse 25 KD.

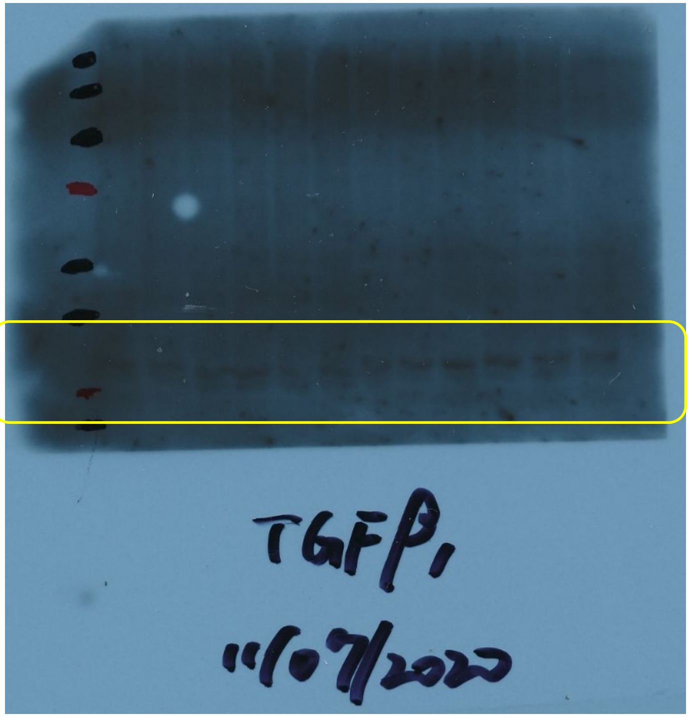

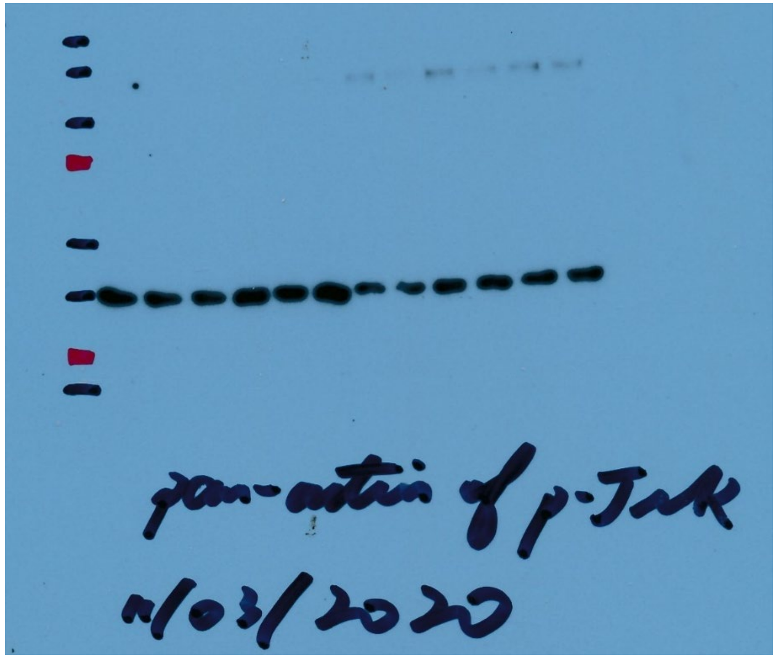

Fig. 7F

Membrane #1

Dr. Wei Li Lab---Western Blot working sheet

63KD

Data:	8/22/2020						Investigator	Liang Hong						
Aim	MMP-2 expression in PC & C2 treated with TNF-α, serum free medium for 0.5h, 2h													
Gel Concentration:	7%													
1st antibody:	MMP-2, mouse, Santa Cruz			Concentration:	1:500			Buffer:	5% skimmed milk					
1st antibody:	anti-mouse HRP			Concentration:	1:2000			Buffer:	5% skimmed milk					
Lane 1	Lane 2	Lane 3	Lane 4	Lane 5	Lane 6	Lane 7	Lane 8	Lane 9	Lane 10	Lane 11	Lane 12	Lane 13	Lane 14	Lane 15
Sample Buffer	Marker 5µl	PC Control	PC 0.5h	PC 2h	C2 Control	C2 0.5h	C2 2h	Marker 2µl	Sample Buffer					

kit: pico pluse → maximum sensitive

loading Control: pan-actin 1:10000

8/23/2020 Strip for CTGF (Connective tissue growth factor, hypertrophic chondrocyte-specific gene product ~~24~~ or HCS24) 38KD Mouse (1:500) Santa Cruz Pico → Maximum Sensit

⇓ 8/24/2020

TYMP 1st Ab (without strip) Abcame (180783) 1:1000
50KD 2nd Ab (1:3000) Rabbit (8/25/2020)

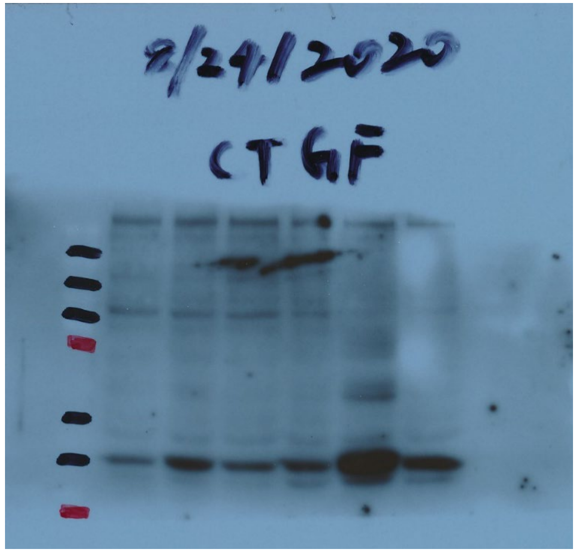

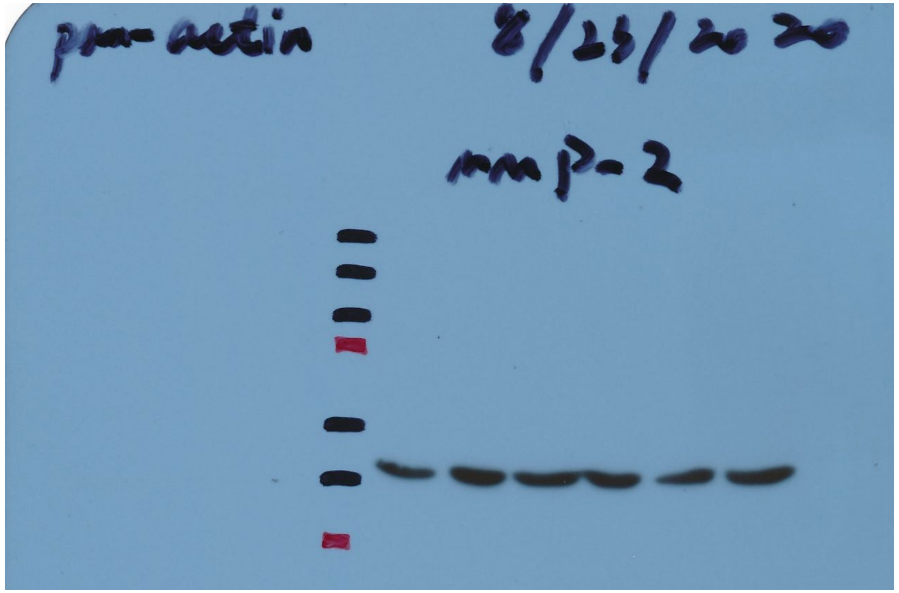

Fig. 7G

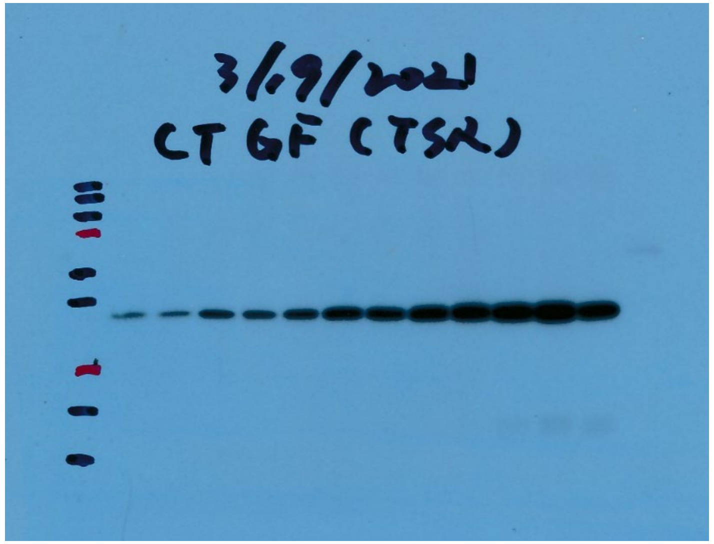

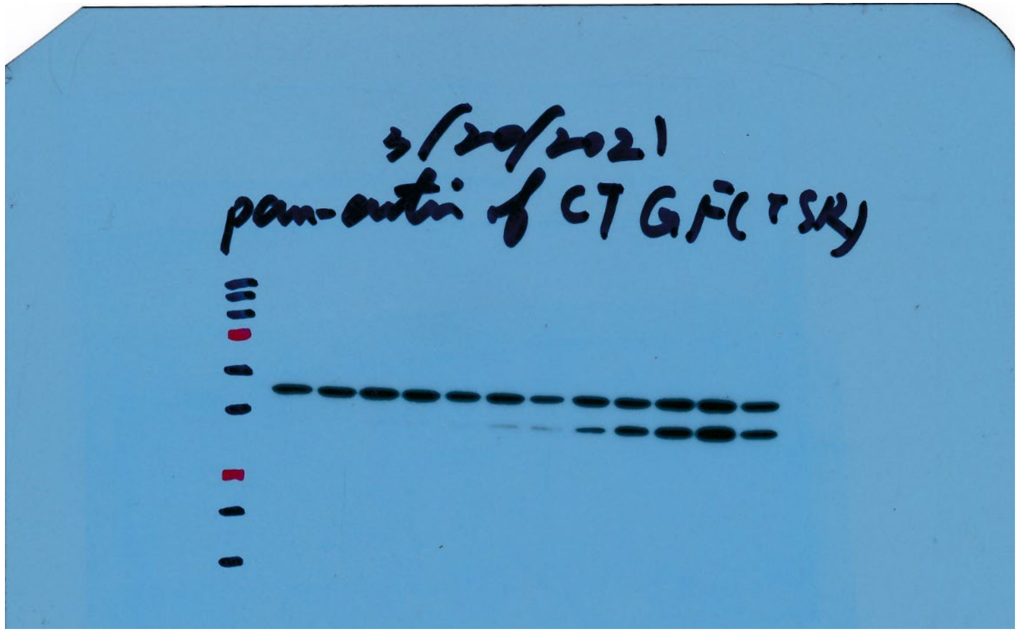

Fig. 7H

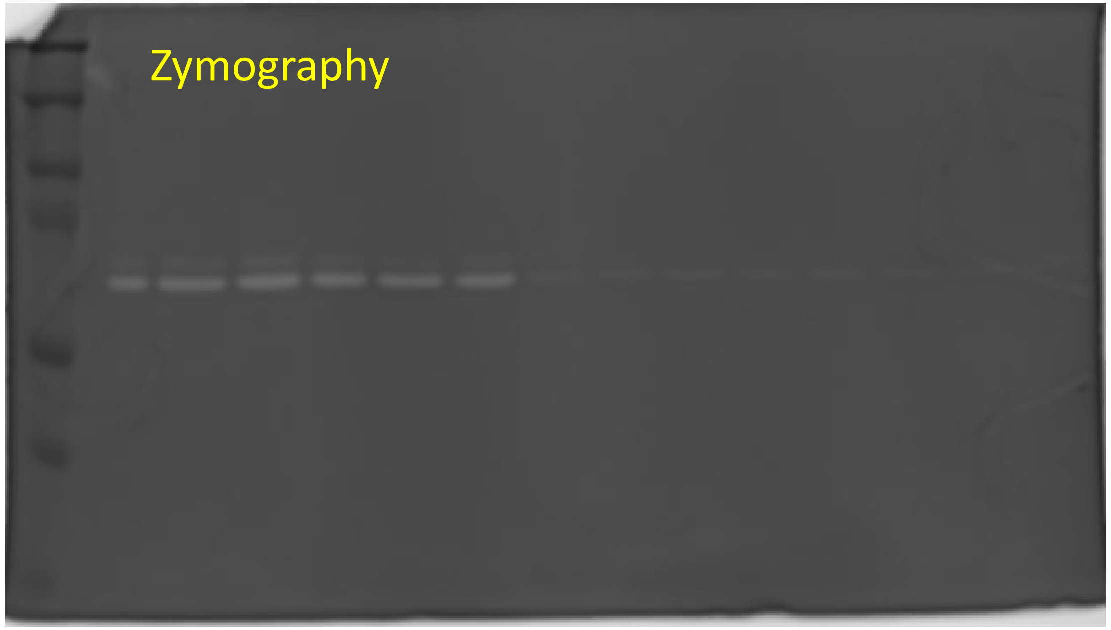

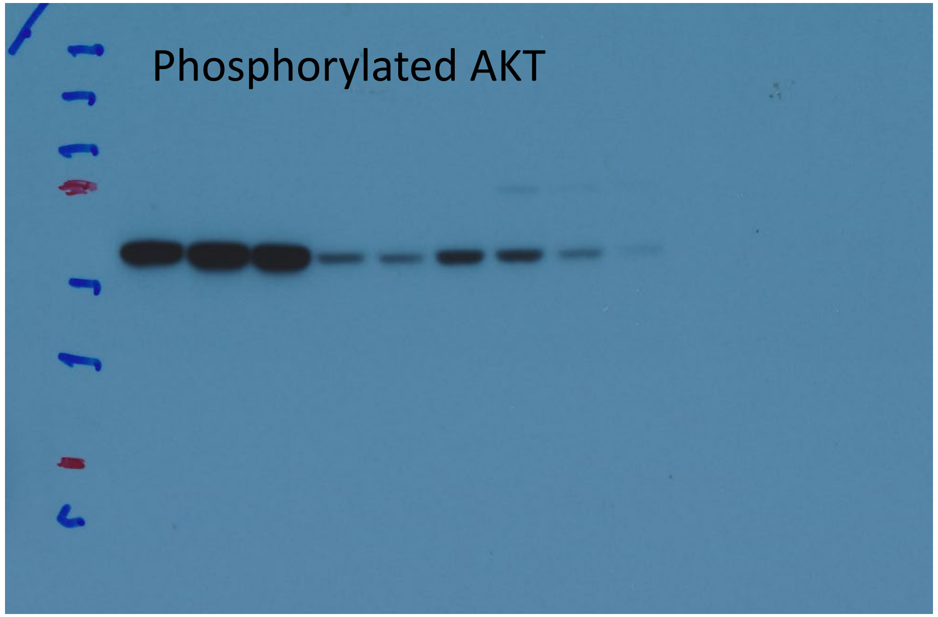

Pan-AKT

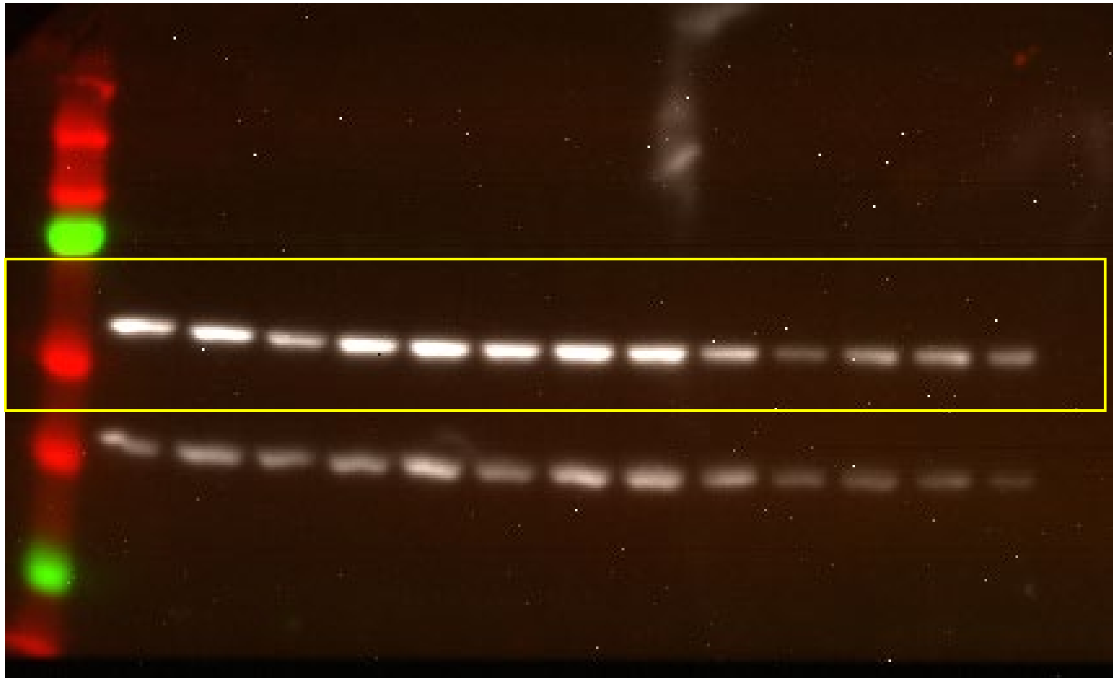

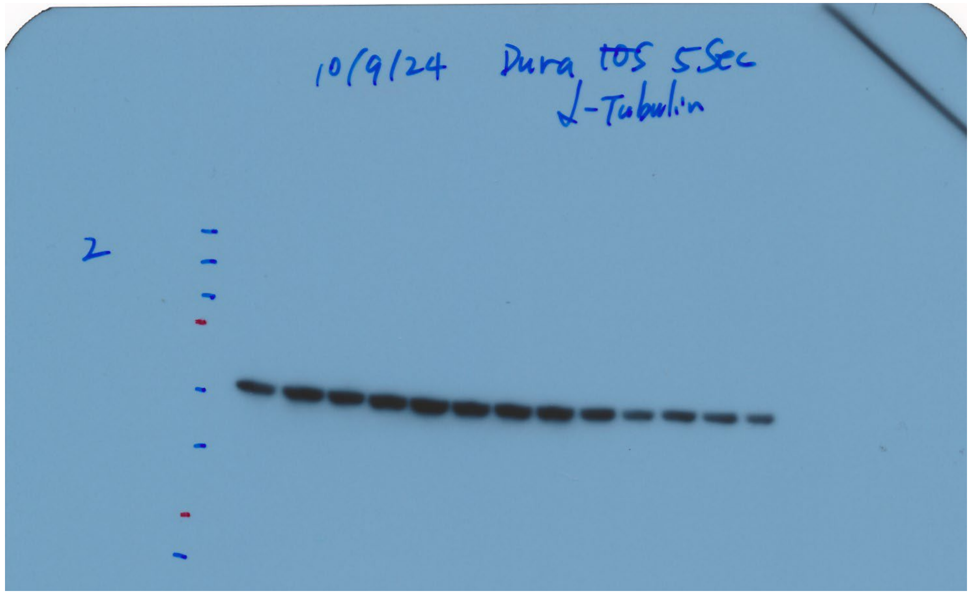

Fig. 7I

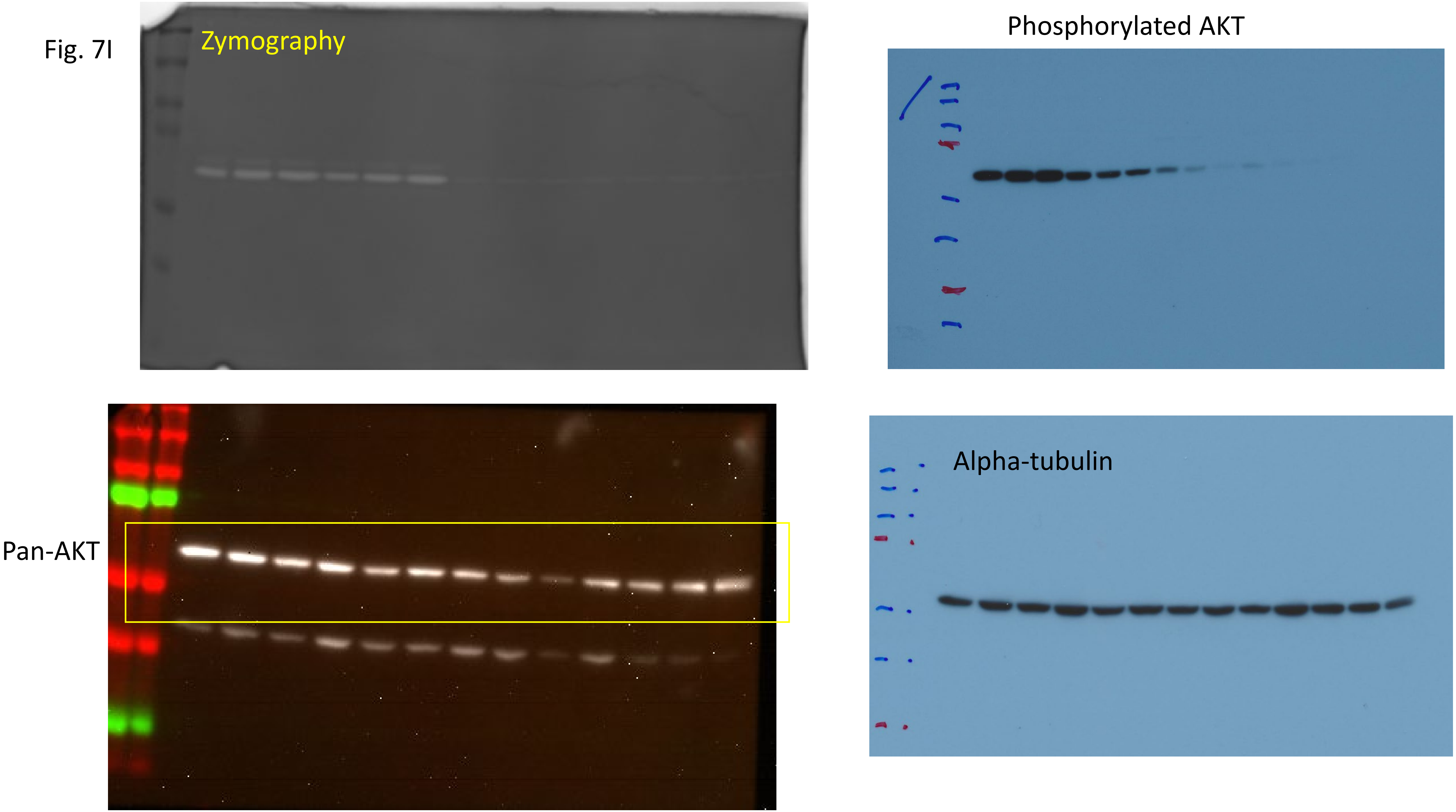

Supplemental Figure 12. A.

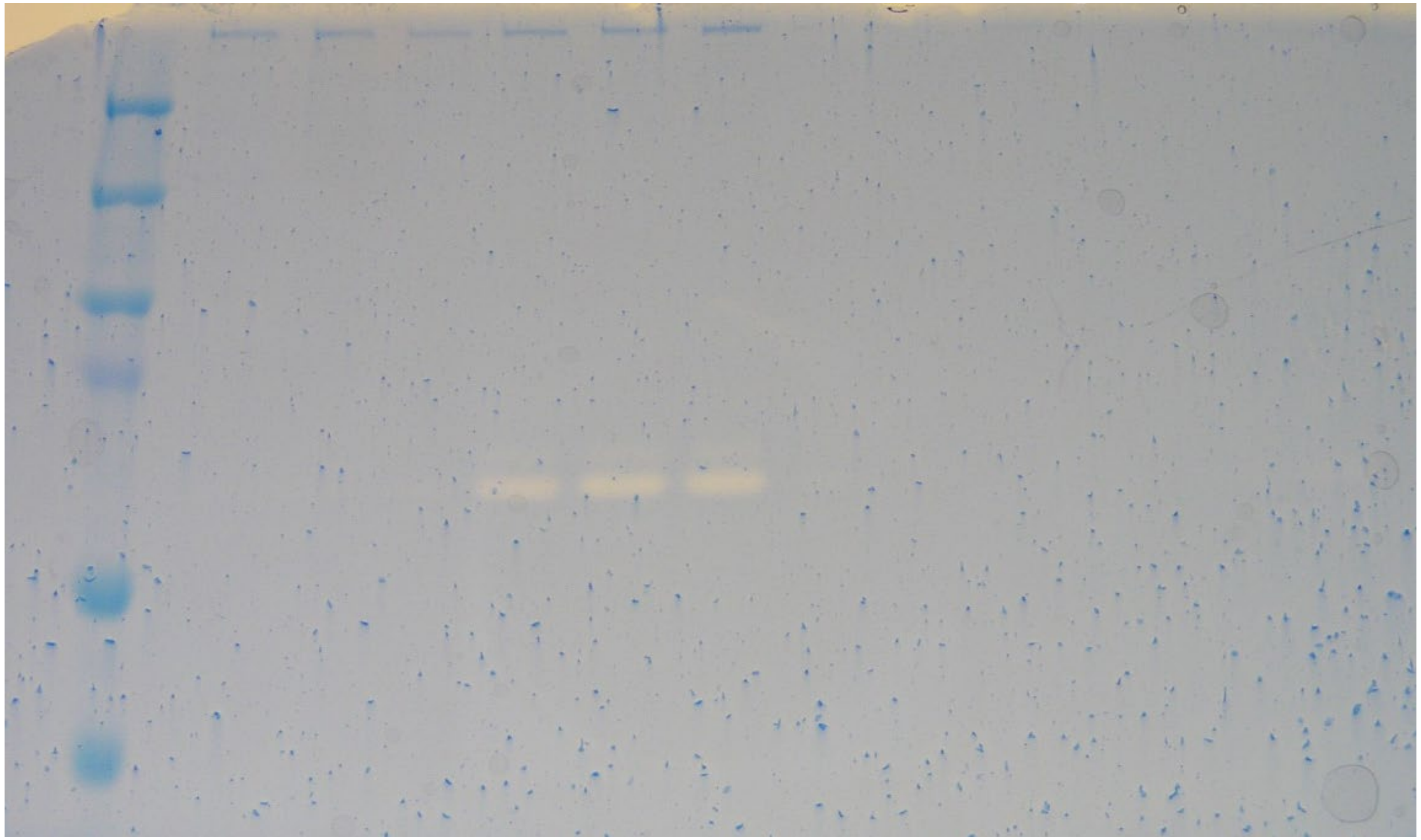

Supplemental Figure 12. B.

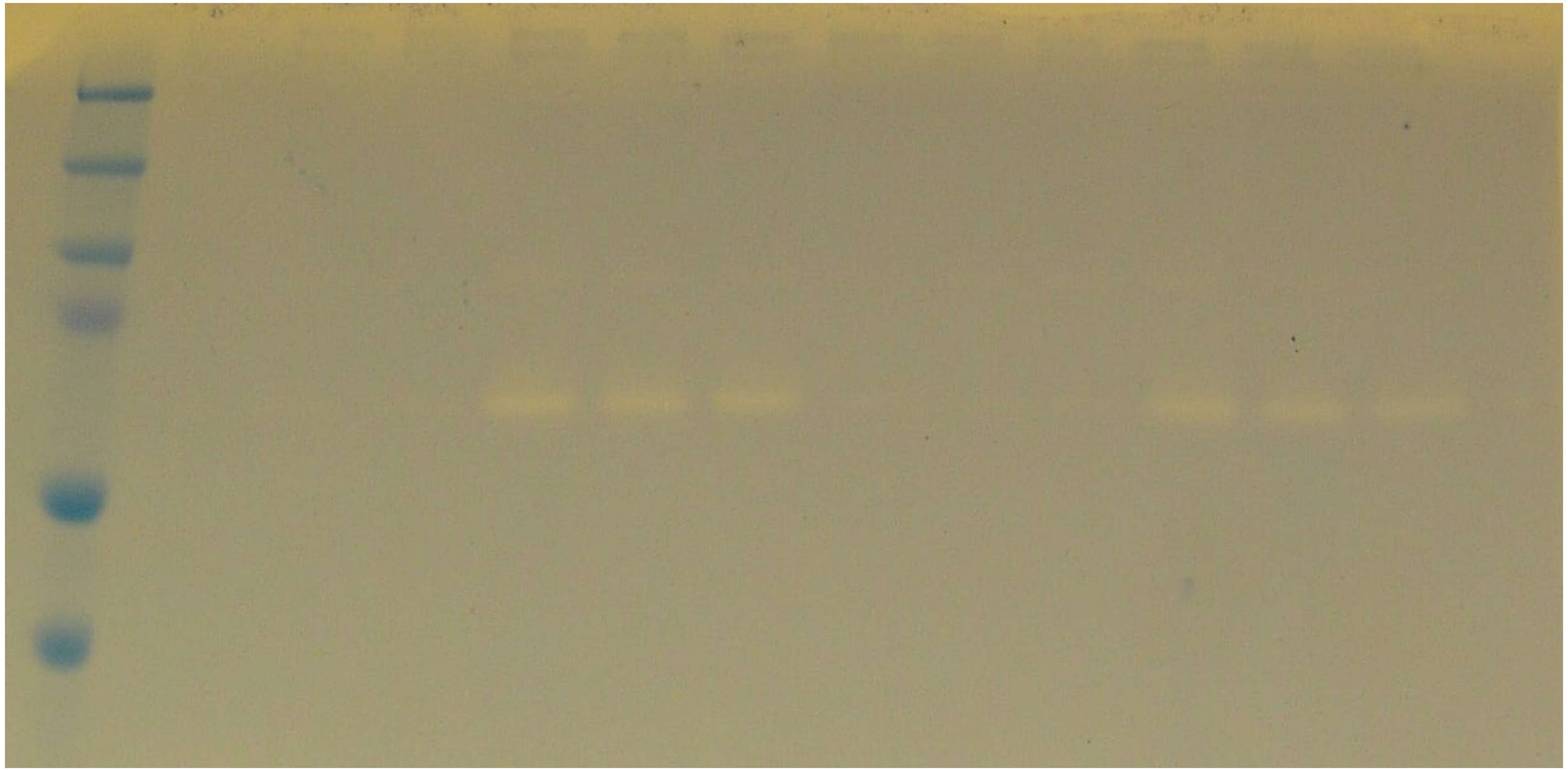

Supplemental Figure 17

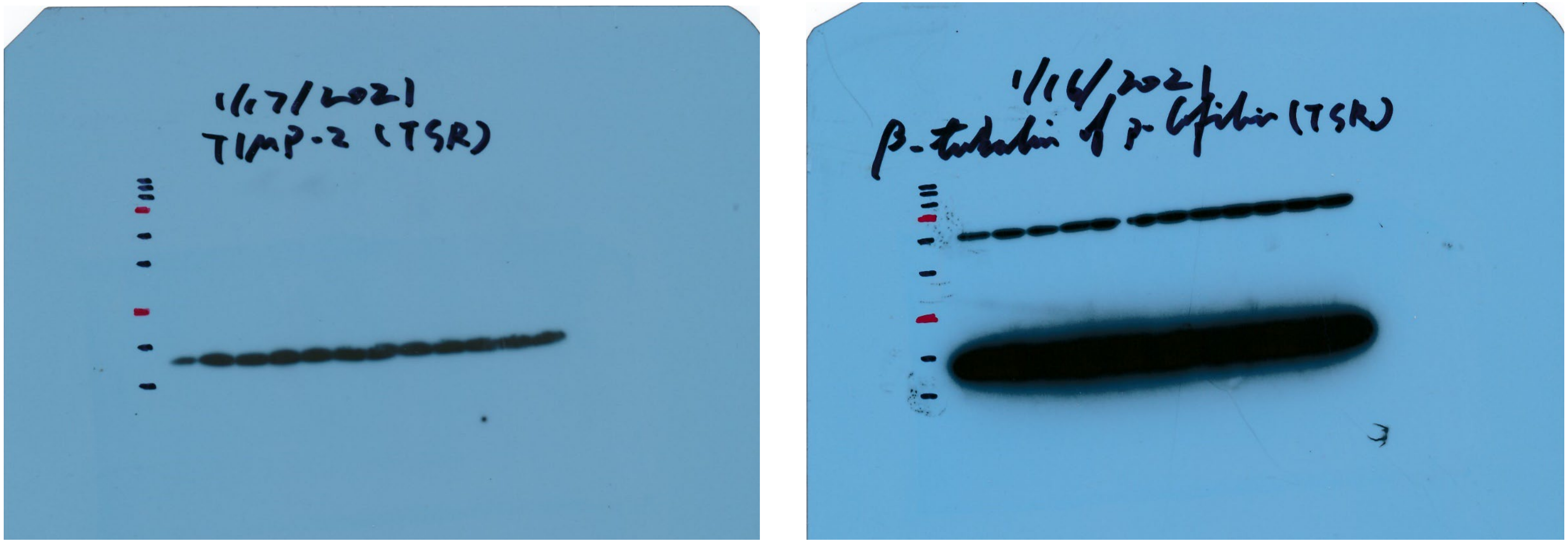

Supplement: Supporting Information 3 — Figure S1. Flow chart of the proposed study. Figure S2. Histochemical staining images were scored based on staining intensity. We adopted a 6-point scale system, and representative images with score assignments are provided. Scale bar = 250 μm. Figure S3. TYMP expression is increased in the human AAA vessel wall. The human AAA vessel wall and healthy control aorta vessel wall (Con) were sectioned and double stained for TYMP and α-SMA. Nuclei were stained with DAPI. Scale bar = 200 μm. Figure S4. Melting curve analysis was performed to confirm the amplicons of human thymidine phosphorylase (TYMP) and glyceraldehyde 3-phosphate dehydrogenase (GAPDH). Figure S5. A. WT and Tymp−/− mice were fed a Western diet starting at 4 weeks of age for 8 weeks. Mouse body weight was monitored weekly until the implantation of the Alzet osmotic mini pump. B. Whole blood was drawn via inferior vena cava puncture at the time of sacrifice, and plasma was isolated by centrifugation using 0.109 M sodium citrate as an anticoagulant (1:9). Plasma triglyceride levels were measured using the triglyceride (TG) Colorimetric Assay Kit (EEA028, ThermoFisher Scientific). Figure S6. Necropsy of mice died after Ang II perfusion. Figure S7. Thymidine phosphorylase (TYMP) deficiency reduced the prevalence of abdominal aortic aneurysm (AAA) formation in mice. Mice were treated as mentioned in Figure 2. A Inner diameter of the abdominal aorta in the diastolic phase. B Inner diameter of the abdominal aorta in the systolic phase. Data are shown as mean ± SEM. N = 12 in WT and 11 in Tymp−/− groups for both A and B. One-way ANOVA was used to determine the time-dependent changes. C Representative echo images in the diastolic phase. D Representative echo images in the systolic phase. Figure S8. A receiver operating characteristic (ROC) analysis using the data from Figure 2(g) (abdominal aorta diameter), with the Tymp−/− group as the control and the WT group as the test group. Figure S9. Thymidine phosphorylase [file 1129181.f3.zip › Uncut original images of WB and Zymography-HL.pdf]
